# Traditional Chinese Medicine Injections Combined With Antihypertensive Drugs for Hypertensive Nephropathy: A Network Meta-Analysis

**DOI:** 10.3389/fphar.2021.740821

**Published:** 2021-10-22

**Authors:** Zhe Chen, Yingying Peng, Fengwen Yang, Xiaoyu Qiang, Yong Chen, Yongjie Chen, Lujia Cao, Chunxiang Liu, Junhua Zhang

**Affiliations:** ^1^ Evidence-based Medicine Center, Tianjin University of Traditional Chinese Medicine, Tianjin, China; ^2^ First Teaching Hospital of Tianjin University of Traditional Chinese Medicine, Tianjin, China; ^3^ National Clinical Research Center for Chinese Medicine Acupuncture and Moxibustion, Tianjin, China; ^4^ Graduate School, Tianjin University of Traditional Chinese Medicine, Tianjin, China; ^5^ Department of Epidemiology and Statistic, School of Public Health, Tianjin Medical University, Tianjin, China

**Keywords:** traditional Chinese medicine injections, antihypertensive drugs, hypertensive nephropathy, network meta-analysis, randomized controlled trial

## Abstract

**Background:** Hypertension, a risk factor for cardiovascular events, is often associated with chronic kidney disease. This is called hypertensive nephropathy (HN), which negatively affects physical fitness and body mass, leading to economic burden. Traditional Chinese medicine injections (TCMIs) are common traditional Chinese-patent medicine preparations in China. There was a lack of evidence to prove which TCMIs combine with ADs (TCMIs+ADs) may be a therapeutic option for HN. Thus, we systematically reviewed the efficacy and safety of various TCMIs + ADs in patients with HN.

**Methods:** We conducted a comprehensive search of PubMed, Embase, Cochrane Library, Web of Science, China National Knowledge Infrastructure, Wanfang Data Knowledge Service Platform, and VIP information resource integration service platform databases for relevant Chinese- and English-language randomized controlled trials (RCTs) published from database inception until May 2021. Literature screening, data extraction, and quality assessment was performed by two reviewers independently but using the same criteria. We performed the effect modeling to analyze the data for all outcomes and ranked each intervention using the P-score. Furthermore, sensitivity analysis, meta-regression, and funnel plots were used to test the stability, heterogeneity, and publication bias, respectively.

**Results:** We included 69 RCTs with 6373 patients and including six TCMIs + ADs. Network analysis indicated that the ginkgo leaf extract and dipyridamole combined with ADs (GLED + ADs) was the most efficacious in terms of 24-h urinary protein excretion [mean difference (MD) = −0.70, 95% confidence interval (CI): −0.82 to −0.58; P-score = 1] and systolic blood pressure (MD = −12.95, 95% CI: −21.03 to −4.88; P-score = 0.88), whereas the salvianolate combined with ADs (SA + ADs) showed the highest effectiveness for diastolic blood pressure (MD = −6.88, 95% CI: −10.55 to −3.21; P-score = 0.9). Based on the combined P-score of network meta-analysis results (88% and 85.26%) and sensitivity analysis results (72% and 71.54%), the biplots showed that the GLED + ADs was the most efficacious intervention in all TCMIs + ADs for primary outcomes, followed by the SA + ADs and sodium tanshinone IIA sulfonate combined with ADs (STS + ADs). There was no significant difference in terms of safety between TCMIs + ADs and ADs alone.

**Conclusion:** Of all the TCMIs + ADs, GLED + ADs, SA + ADs, and STS + ADs may demonstrate a higher efficacy than ADs alone for HN. Weighing with the potential benefits and limitations in methodology, potential heterogeneity and outcomes, we should use various TCMIs with caution in clinical practice. Nevertheless, additional high-quality RCTs are warranted and future research should focus on the clinical value of core outcomes to confirm the effectiveness and safety of TCMIs for HN.

**Systematic Review Registration**: clinicaltrials.gov, identifier CRD42020205358

## Introduction

Hypertension is a well-established independent, significant risk factor for high cardiovascular disease (CVD) morbidity and mortality globally (Collaborators 2018). According to a 2017 American College of Cardiology/American Heart Association report, hypertension prevalence and recommended antihypertensive medication dosage were on the rise, where the increasing trend of the latter was slightly lower than that of the former (Muntner et al., 2018). Hypertension is frequently associated with chronic kidney disease (CKD): CKD primarily results from long-term hypertension, and it can lead to kidney damage as well as multiple clinical symptoms that worsen physical fitness and body mass (Byrd and Brook, 2019). In addition to unstable blood pressure (BP), hypertensive patients with CKD always have impaired renal function (e.g., abnormalities in glomerular filtration rate, urinary albumin, urinary sediment, and kidney histology) (Castillo-Rodriguez et al., 2019). Hypertension and CKD with multiplicative and cumulative effects are concurrently primary risk factors for cardiovascular events and mortality (Navaneethan et al., 2015; Liu et al., 2020). Therefore, the increasing emphasis on hypertension with CKD, or hypertensive nephropathy (HN), and concomitant diseases during treatment warrants considerable attention.

The therapeutic regimens of patients with HN focus on BP control by using angiotensin-converting enzyme (ACE) inhibitors (ACEIs) or angiotensin receptor blockers (ARBs). These drugs slow kidney disease progression and maintain the BP at less than 130/80 mmHg in patients with relatively severe proteinuria (Whelton et al., 2018). Most hypertensive patients with CKD globally die of the consequences of cardiovascular complications (van der Zee et al., 2009). Thus, in clinical management, long-term rational antihypertensive drugs (ADs) are prescribed to lower BP within the optimal range and thus effectively control the complications of CVD and the potential fatality risks during the pathogenesis of the disease (van der Zee et al., 2009; Whelton et al., 2018). In a population of hypertensive patients without other serious diseases, intensive BP control could reduce cardiovascular events and cardiovascular mortality (Williamson et al., 2016). However, in patients with HN, intensive BP control can significantly reduce systolic BP (SBP), thus increasing mortality risk and worsening kidney injury (Weiss et al., 2015; Stompór and Perkowska-Ptasińska, 2020). Based on the complexity of the pathogenesis and chronic course of hypertension, the affected patients’ BP must be appropriately adjusted to achieve the expected optimal individualized treatment effects based on the overall disease status (Whelton et al., 2018; Stompór and Perkowska-Ptasińska, 2020). The various treatment guidelines for hypertension differ slightly in their methods and backgrounds, but they all have a similar clinical therapeutic purpose (McCormack et al., 2019). This purpose is to guide clinical professionals when choosing the most effective treatment strategies of personalized medication for patients with hypertension and to improve the awareness of personalized treatment and the related new trends among patients (Byrd and Brook, 2019; Melville and Byrd, 2019).

Traditional Chinese medicine (TCM) fully embodies an overall combination of multiple therapeutics and individualized treatments in the healthcare system. Its effects have been confirmed through over thousands of years of clinical practice in China (Yuan 2021). TCM, which includes natural Chinese medicine extracts and various natural Chinese medicine combination preparations, has received increasing attention for CVD treatment. It has become the supplementary or alternative approach for primary and secondary preventions of CVD (Hao et al., 2017; Zhao et al., 2020). TCM can reduce inflammation and inhibit oxidative stress and thus alleviate the HN damage caused by hypertension and renal injury through multiple signal pathways (Ding et al., 2015; Yan et al., 2018; Guan et al., 2020). In China, clinicians widely prescribe TCM injections (TCMIs), a type of traditional Chinese-patent medicine registered in National Medical Products Administrations, in combination with ADs to treat HN. TCMIs can effectively alleviate hypertension-induced renal injury by inhibiting kidney injury molecule-1 expression and downregulating myoglobin expression (Owoicho Orgah et al., 2018).

The randomized controlled trials (RCTs) and systematic reviews reported thus far have evaluated and verified the clinical efficacy of certain TCMIs for HN (Sun et al., 2012; Xu et al., 2019; Li et al., 2020). Base on this current evidence, it is thus impossible to recommend the TCMIs with better clinical utility from the various TCMIs for HN and solve the dilemma of clinical decisions that have been evaluated simply by analyzing certain TCMIs. The rationale for conducting this network analysis is that previous published systematic reviews have focused on the treatment of HN with a single TCMI (Sun et al., 2012; Xu et al., 2019; Li et al., 2020), but there were lacked the evidence of compare various TCMIs for HN. Therefore, this study systematically evaluated and compared the clinical efficacy and safety of several TCMIs combined with ADs (TCMIs + ADs) used for HN treatment for the first time. The purpose of this study is to provide sufficient clinical evidence for evidence-based medicine of traditional Chinese medicine with the most appropriate evidence-based medicine methods, and can provide some reference value for the decision-making of clinical guidelines and clinical practice of traditional Chinese medicine regarding the use of TCMIs + ADs for HN.

## Methods

### Protocol and Registration

The protocol of this network meta-analysis was registered and published in the International Prospective Register of Systematic Reviews (PROSPERO), under the number CRD42020205358. We followed the Preferred Reporting Items for Systematic Reviews and Meta-Analyses (PRISMA) and its protocols and the PRISMA-extension statement for network meta-analysis to report the current results (Moher et al., 2009; Hutton et al., 2015; Shamseer et al., 2015). Next, we performed some regulative amendments related to statistical methodology during the study process. Additional details of protocol amendments are listed in Supplementary File S1.

### Eligibility Criteria

We included eligible Chinese- and English-language RCTs that examined the effect and safety of various TCMIs + ADs for HN (i.e., high BP with CKD). The clinical diagnostic criteria of patients with HN was performed following the clinical guideline (Muntner et al., 2018; Whelton et al., 2018). No race, age, and sex restrictions were applied for patient recruitment. Moreover, no intervention time, geographic location, and disease course limitations were applied for study inclusion. We only considered RCTs including TCMIs + ADs and ADs, and AD administration needed to be consistent within the same RCT. The concerned treatment interventions were eligible if TCMIs were the patent preparations approved by the State Drug Administration for hypertension and HN. No restrictions were placed on the included RCTs or on the AD types, including ARBs, ACEIs, and calcium channel blockers (CCB).

We excluded studies if there were serious complications and other life-threatening conditions. We also excluded studies with missing data, repeated research, no full-text articles, and error data. Ineligible studies also included case reports, cohort studies, and animal and cell experiments as well as those comparing treatments other than TCMIs and ADs, such as non-ADs, acupuncture, massage, and decoction.

### Search Strategy

We searched PubMed, Embase, Cochrane Library, Web of Science, China National Knowledge Infrastructure, Wanfang Data Knowledge Service Platform, and VIP information resource integration service platform databases for relevant RCTs published from database inception until May 2021. We used combination of keywords (including MeSH keywords around “traditional Chinese medicine injections” and “hypertensive nephropathy”) designed through specialist consensus. To ensure the comprehensiveness of the current systematic review and network meta-analysis, we also researched clinicaltrials.gov and Chinese clinical trial registry for unpublished trials and gray literature and scanned the reference lists of included studies and published systematic reviews in the concurrent phase for potential studies. The detailed search strategy including search terms has been reported in Supplementary File S2.

### Outcomes

Our primary outcomes were designed to follow the crucial features of HN. Thus, we considered 24-h urinary protein excretion (UPE) and microalbuminuria (mALB) as the powerful indicators. The included studies did not fully report the changes from baseline of absolute mALB in various interventions. Therefore, we could not compare all mALB interventions in the network meta-analysis. Finally, our primary outcome of renal function was 24-h UPE, which has been used as the common method to measure urinary protein relying on 24-h urine collection. In addition, systolic BP (SBP) and diastolic BP (DBP) were considered the primary outcomes to represent BP change. The secondary outcomes included the changes from baseline of absolute mALB, absolute serum creatinine (SCR), absolute blood urea nitrogen (BUN), absolute β2-microglobulin (β2-MG), absolute creatinine clearance rate (CCR), and adverse events (AEs).

### Literature Screening and Data Extraction

Two investigators (YC and XQ) independently screened all studies by title, abstract, and full-text according to the prespecified eligibility criteria. To assess study eligibility, investigators also independently extracted all data from relevant literature (basic information, binary outcomes, and dichotomous outcomes) from the retrieved databases and other available sources to extract additional comprehensive information according to the same criteria. We also contacted the authors and the publishing organizations if some outcomes were missing and demonstrated irregularities. Any disagreements were resolved via discussion and consensus from a third researcher, if necessary.

In detail, we extracted the following items: study characteristics (author, year of publication, research type, country, and time of intervention), participant characteristics (sample size, age, gender, and course of disease), description of interventions (name, drug class, pharmaceutical company, dose, and usage mode), and description of outcomes that changed before and after treatment.

### Risk of Bias Assessment

The quality assessment of the included studies was conducted by two investigators (YJC and LC) using the Cochrane risk of bias (RoB) tool (Higgins et al., 2011). Any discrepancies were resolved via consensus and discussion by a panel of researchers (FY, CL, and JZ) with field experts. RoB items were in the following domains: random sequence generation, allocation sequence concealment, blinding (participants, personnel, and outcome assessment), incomplete outcome data, selective outcome reporting, and other bias. Low, unclear, and high RoBs were used to assess each RoB item criterion.

### Statistical Analysis

We measured dichotomous outcomes (i.e., AEs) as odds ratios (ORs) with 95% confidence intervals (CIs) and continuous outcomes (primary and secondary outcomes) as mean differences (MDs) with 95% CIs. We adopted the frequentist random-effect model through a graph-theoretical approach (Rücker 2012). The estimator was based on weighted least-squares regression. The between-study heterogeneity was estimated using the DerSimonian–Laird method. The heterogeneity was quantified using Cochran’s Q, and the interventions were ranked using P-score. All comparisons were directly performed with ADs, and thus, we did not use the node-splitting approach to calculate the inconsistencies among the direct and indirect evidence.

To decrease the potential modifiers that possibly affect the results, we performed the univariate meta-regression to resolve possible heterogeneity sources. We selected multiple covariants to verify potential heterogeneity, involving the use of ADs (combination of ADs and ADs alone), types of ADs (ARBs, ACEIs, CCBs combined with ARBs or ACEIs, and multiple ADs), sample size, male sex, mean age, and disease and treatment courses. Predictive mean matching imputations were used to supplement and resolve for the missing data on covariants in the regressors. When 10 or more studies were included in the network, we assessed the publication bias using comparison-adjusted funnel plots and Egger’s and Begg’s tests.

To assess reliability and robustness, we performed sensitivity analyses for primary outcomes related to the recommended ADs for HN patients at different CKD stages. High Blood Pressure Clinical Practice Guideline (Whelton et al., 2018) recommends different treatments for different stages of HN: ARBs or ACEIs for in stage 3 or higher CKD or stage 1 or 2 CKD with t-albuminuria (albumin-to-creatinine ratio of ≥300 mg/ d or ≥300 mg/ g). Subsequently, we eliminated multiple ADs without ARB or ACEI to verify the robustness and reliability of our adjusted results in using ARBs or ACEIs by using sensitivity analysis.

All analyses were performed using R (version 4.0.5), and the RoB graph was generated using RevMan (version 5.3).

## Results

### Literature Review

**FIGURE 1:**
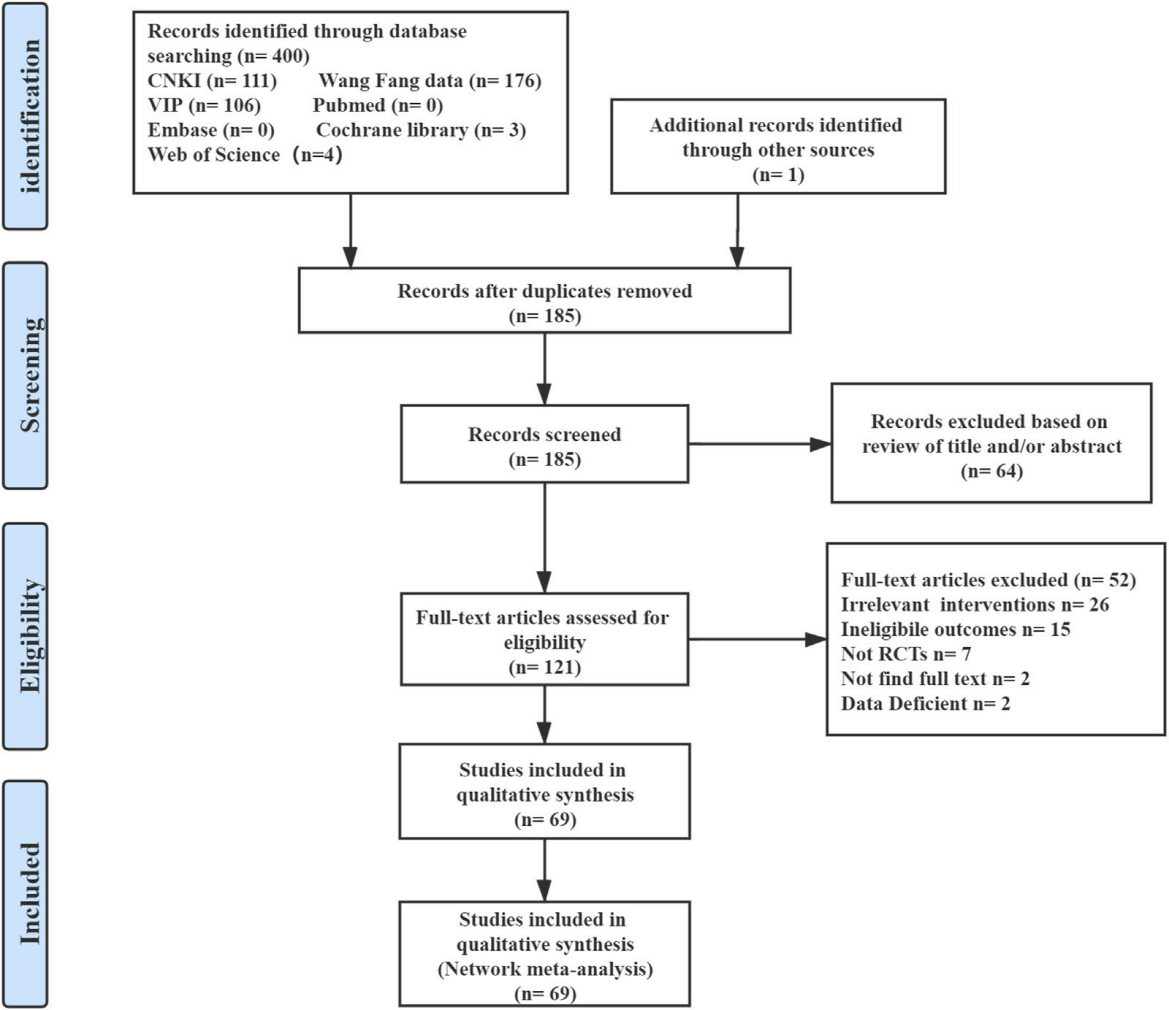
Summary of evidence search and selection.

The results of this network meta-analysis generated 400 citations from the seven databases published between database inception and May 2021. In total, we screened the titles and abstracts of 185 articles included after the removal of the duplicates. We reviewed the full-text of the remaining 121 potentially eligible articles, of which 69 RCTs were deemed eligible. The detailed selection process is shown in Figure 1.

### Description of Included Studies

 The 69 RCTs included 6373 patients with HN enrolled to receive seven interventions (3214 patients under six different TCMI + AD treatments and 3159 under AD treatment alone). The TCMIs + ADs included ADs combined with salvianolate (SA; SA + ADs), danhong (DH; DH + ADs), breviscapine (BRE; BRE + ADs), astragalus (AST; AST + ADs), sodium tanshinone IIA sulfonate (STS; STS + ADs), ginkgo leaf extract and dipyridamole (GLED; GLED + ADs), and ADs (ARB, ACEI, etc.). All 69 studies recruited patients from China. Their sample sizes ranged from 37 to 230 patients, trial mean age ranged from 36.34 to 76.6 years (median = 59.48 years), the male-to-female ratio was 1.4:1, disease course ranged from 2.25 to 17.5 years (median = 9.92 years), and treatment course ranged from 1 to 12 weeks (median = 3.63 weeks). All reference of the included studies are provided in Supplementary File S3. Detailed characteristics of all included studies are listed in Supplementary Table S1. We also revised the dosages of TCMIs and ADs. The detailed chemical characterization of the TCMIs is shown in Supplementary Tables S2, S3.

### Methodological Quality Assessment

Supplementary Figures S1, S2 present the results of the RoB assessment of 69 RCTs for each item. All included studies reported the random assignment, and 24.64% of studies fully reported the specific methods of random sequence generation. Allocation sequence concealment of all included studies was unclear. Only 5.8% of the studies reported participant and personnel blinding. Approximately 60.87% of the RCTs may not have selected the reporting outcomes. In most included RCTs, no data were missing, but other bias was unclear.

## Network Meta-Analysis Results

### Primary Outcomes

**FIGURE 2:**
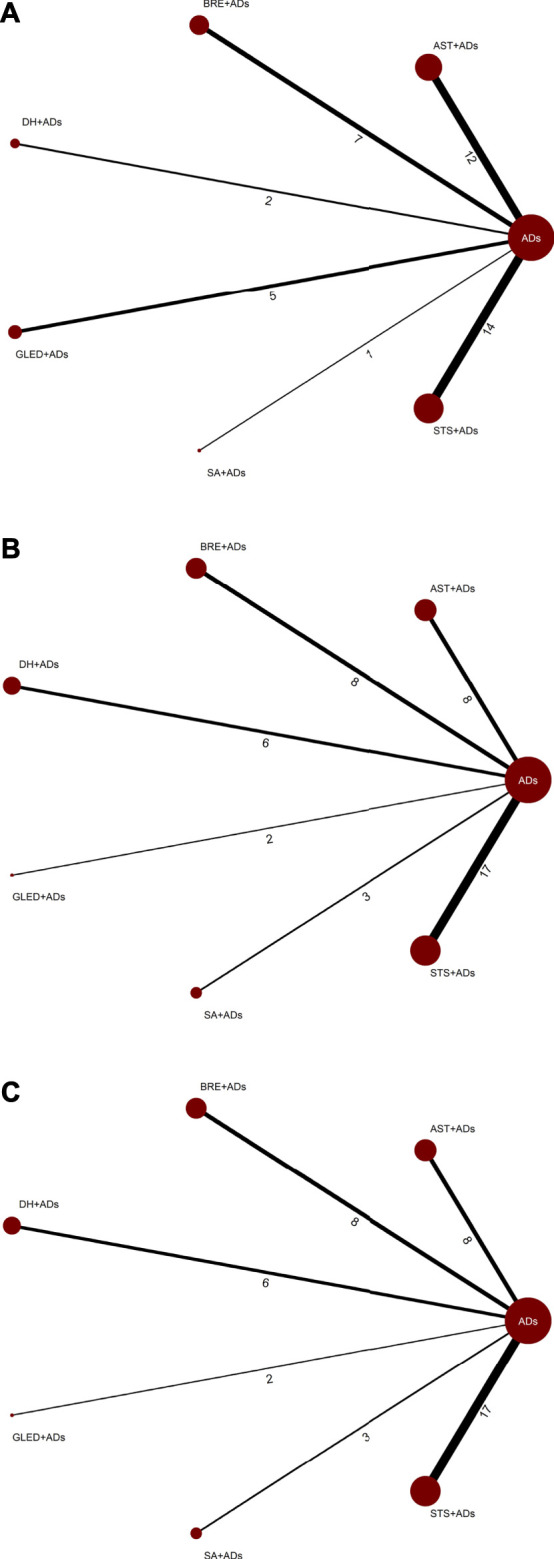
Network plots for primary outcomes. ADs: antihypertensive drugs; SA: salvianolate; DH: danhong; BRE: breviscapine; AST: astragalus; STS: sodium tanshinone IIA sulfonate; GLED: ginkgo leaf extract and dipyridamole. **(A)**: 24-h urinary protein excretion; **(B)**: Systolic blood pressure; **(C)**: Diastolic blood pressure.

The network plots included all interventions for 24-h UPE, SBP, and DBP, and each of the nodes represented the various TCMIs + ADs and ADs, contributing to direct comparisons (Figure 2).

#### 24-Hour UPE

**FIGURE 3:**
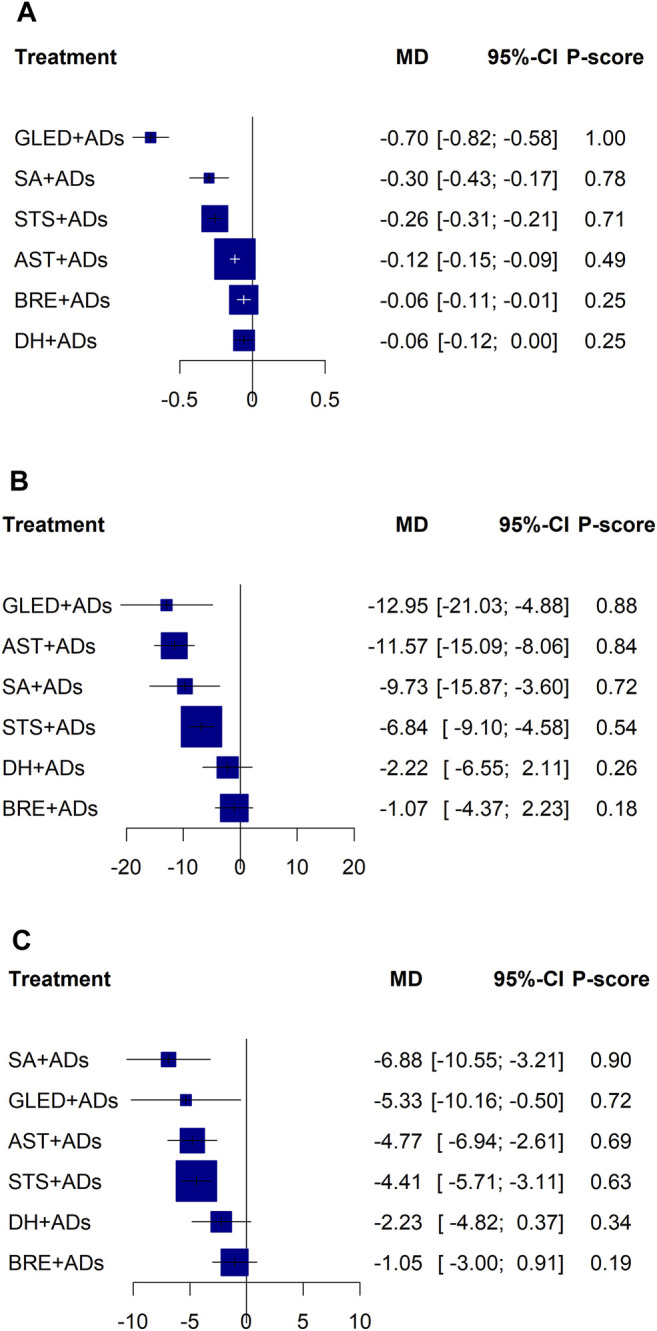
Forest plots for primary outcomes. ADs: antihypertensive drugs; SA: salvianolate; DH: danhong; BRE: breviscapine; AST: astragalus; STS: sodium tanshinone IIA sulfonate; GLED: ginkgo leaf extract and dipyridamole. **(A)**: 24-h urinary protein excretion; **(B)**: Systolic blood pressure; **(C)**: Diastolic blood pressure.

In total, 41 RCTs including 3874 patients reported continuous data for 24-h UPE. Five TCMIs + ADs were more efficacious in improving 24-h UPE than ADs alone: AST + ADs (MD = −0.12, 95% CI: −0.15 to −0.09), BRE + ADs (MD = −0.06, 95% CI: −0.11 to −0.01), SA + ADs (MD = −0.30, 95% CI: −0.43 to −0.17), STS + ADs (MD = −0.26, 95% CI: −0.31 to −0.21), and GLED + ADs (MD = −0.70, 95% CI: −0.82 to −0.58; Figure 3A). According to P-score, the top-ranked intervention was GLED + ADs (1.00), followed by SA + ADs (0.78), STS + ADs (0.71), and AST + ADs (0.49). Network estimates of the pooled effects in the league table (Table 1, top panel) shows that GLED + ADs was the most effective interventions of TCMIs + ADs with a significant positive effect on 24-h UPE.

**TABLE 1:** League table for 24-h UPE.

24-h UPE [mean difference (95% confidence interval)]						
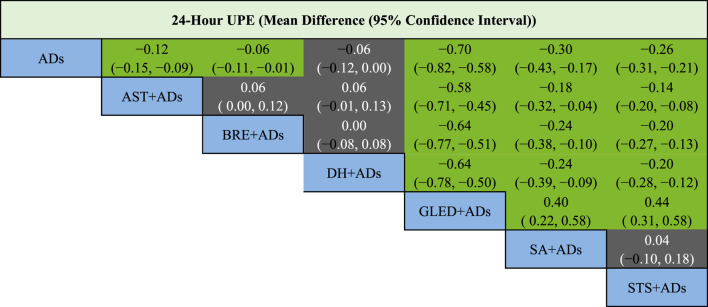

ADs, antihypertensive drugs; AST, astragalus; BRE, breviscapine; DH, danhong; GLED, ginkgo leaf extract and dipyridamole; SA, salvianolate; STS, sodium tanshinone IIA sulfonate; UPE, urinary protein excretion.

Green cells represent significance difference, whereas gray cells represent no significance difference.

#### SBP

In total, 43 RCTs including 4280 patients reported the SBP. Four TCMIs + ADs were more efficacious in controlling SBP than ADs alone: AST + ADs (MD = −11.57, 95% CI: −15.09 to −8.06), SA + ADs (MD = −9.73, 95% CI: −15.87 to −3.60), STS + ADs (MD = −6.84, 95% CI: −9.10 to −4.58), and GLED + ADs (MD = −12.95, 95% CI: −21.03 to −4.88; Figure 3B). According to P-score, the top-ranked intervention was GLED + ADs (0.88), followed by AST + ADs (0.84), SA + ADs (0.72), and STS + ADs (0.54). The league table (Table 2; top panel) shows that GLED + ADs, SA + ADs and STS + ADs were the most effective interventions with a positive effect on SBP.

**TABLE 2:** League table for SBP and DBP.

	SBP [mean difference (95% confidence interval)]						
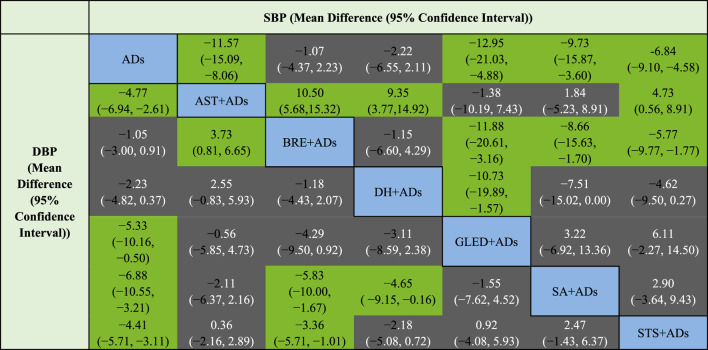

ADs, antihypertensive drugs; AST, astragalus; BRE, breviscapine; DBP, Diastolic blood pressure; DH, danhong; GLED, ginkgo leaf extract and dipyridamole; SA, salvianolate; SBP, Systolic blood pressure; STS, sodium tanshinone IIA sulfonate; UPE, urinary protein excretion.

Green cells represent significance difference, whereas gray cells represent no significance difference.

#### DBP

In total, 43 RCTs including 4280 patients reported the DBP. Four TCMIs + ADs were more efficacious in improving DBP than ADs alone: AST + ADs (MD = −4.77, 95% CI: −6.94 to −2.61), SA + ADs (MD = −6.88, 95% CI: −10.55 to −3.21), STS + ADs (MD = −4.41, 95% CI: −5.71 to −3.11), and GLED + ADs (MD = −5.33, 95% CI: −10.16 to −0.50; Figure 3C). According to P-score, the top-ranked intervention was SA + ADs (0.90), followed by GLED + ADs (0.72), AST + ADs (0.69), and STS + ADs (0.63). The league table (Table 2; bottom panel) shows no significant differences between the top four interventions (SA + ADs, GLED + ADs, AST + ADs, and STS + ADs) of TCMIs + ADs for DBP.

### Sensitivity Analysis of Primary Outcomes

**FIGURE 4:**
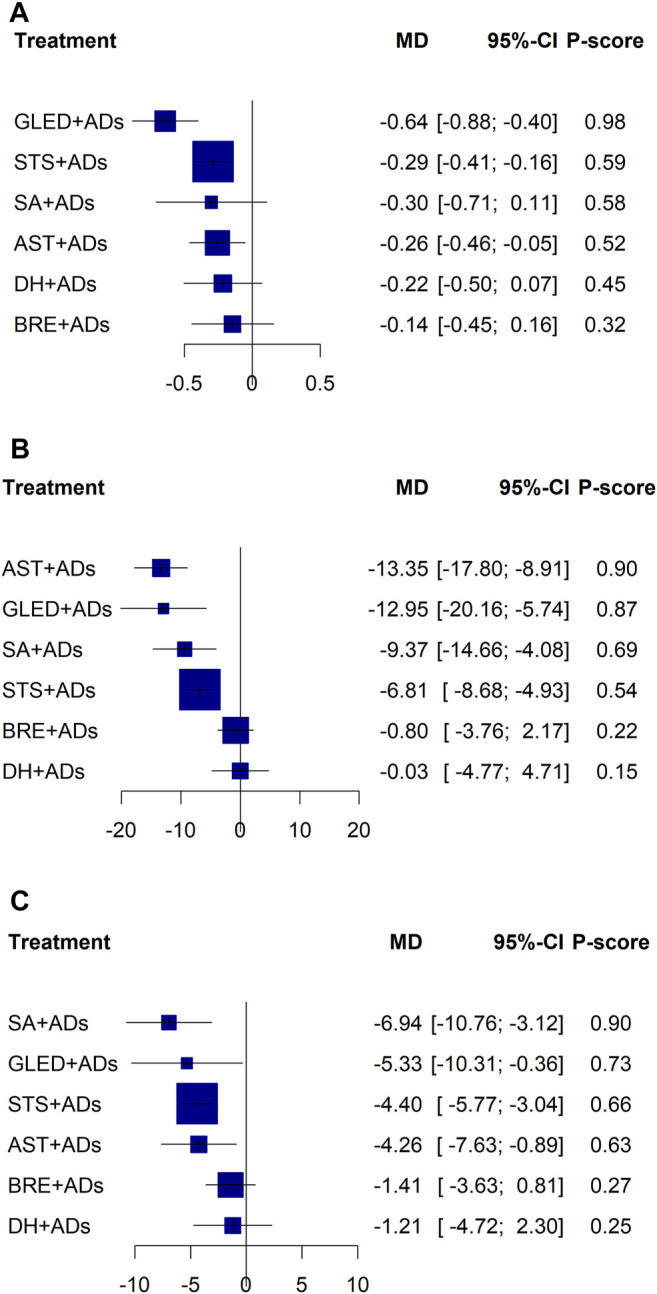
Sensitivity forest plots for primary outcomes. ADs: antihypertensive drugs; SA: salvianolate; DH: danhong; BRE: breviscapine; AST: astragalus; STS: sodium tanshinone IIA sulfonate; GLED: ginkgo leaf extract and dipyridamole. **(A)**: 24-h urinary protein excretion; **(B)**: Systolic blood pressure; **(C)**: Diastolic blood pressure.

We conducted a sensitivity analysis to assess the reliability and robustness of the results according to the recommended ADs (ARBs or ACEIs) with disease progression stages. After the exclusion of other RCTs, the remaining 28, 35, and 35 RCTs reported the 24-h UPE, SBP, and DBP, respectively. Sensitivity analysis results did not show significant deviations compared with the effect estimate results of the original network analysis, and P-scores did not change the probability of ranking substantially (Figure 4).

### Biplot of Primary Outcomes

Based on the P-score of the original network analysis and sensitivity analysis results, we used biplots to combine 24-h UPE with SBP and DBP to determine the best TCMIs + ADs in the network analysis.

**FIGURE 5:**
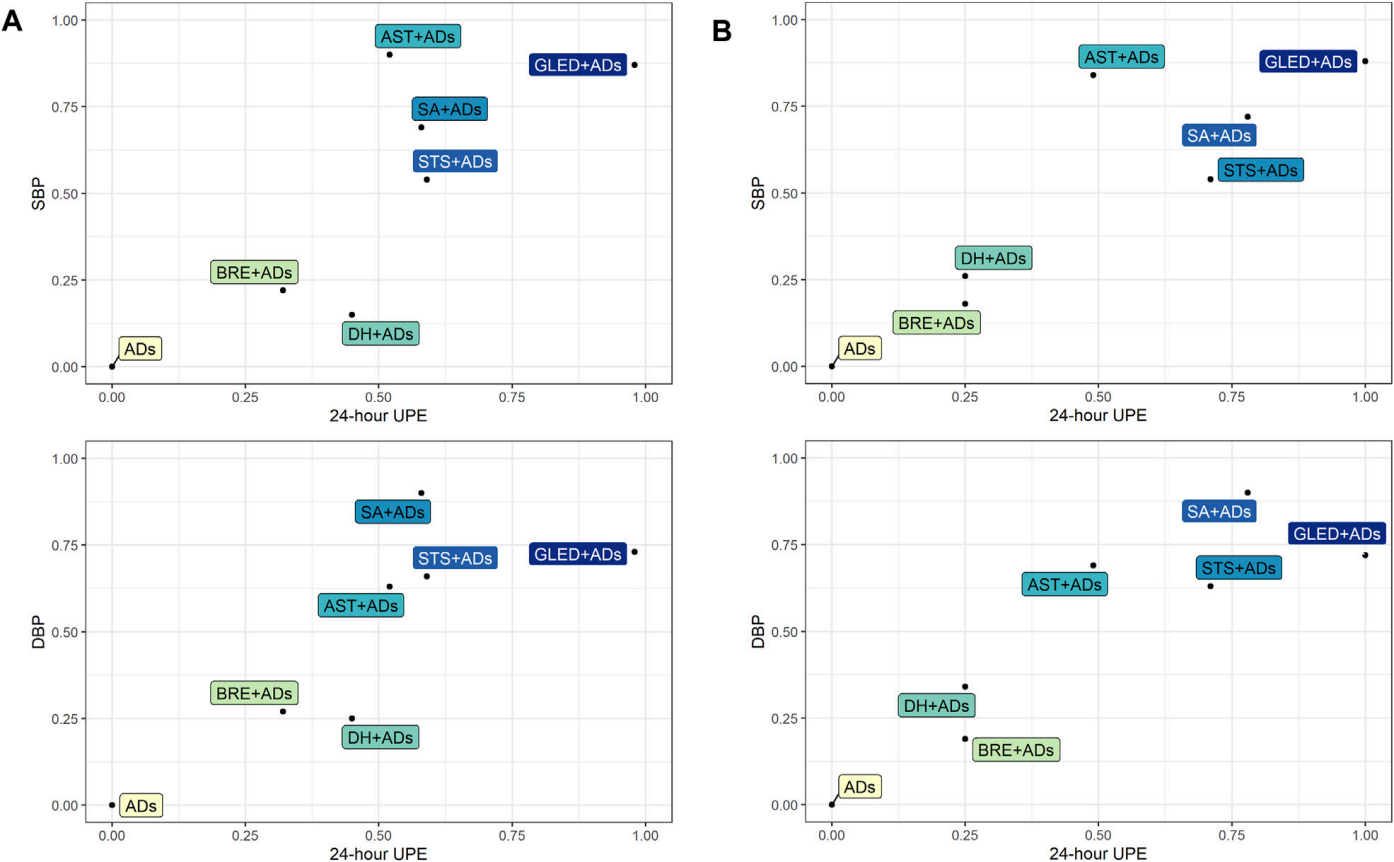
Biplots for primary outcomes. ADs: antihypertensive drugs; SA: salvianolate; DH: danhong; BRE: breviscapine; AST: astragalus; STS: sodium tanshinone IIA sulfonate; GLED: ginkgo leaf extract and dipyridamole. **(A)**: Original network analysis results; **(B)**: Sensitivity analysis results.

The biplot results confirmed that GLED + ADs may the best interventions with the highest combined P-score in the original network analysis results (88 and 85.26%, respectively) and sensitivity analysis results (72 and 71.54%, respectively) among the eligible interventions (Figure 5 and Supplementary Table S4).

### Secondary Outcomes

#### mALB

In total, 20 RCTs reported mALB in 1775 patients randomized to receive AST + ADs, BRE + ADs, DH + ADs, SA + ADs, STS + ADs, and ADs alone (Supplementary Figure S3). Five TCMIs + ADs have significant differences compared with ADs, and BRE + ADs had the best P-score (1.00; Supplementary Figure S4). Network meta-analysis results showed that BRE + ADs have significant differences compared with other TCMIs + ADs for mALB (Supplementary Table S5).

#### SCR

Network plots included all interventions showed that 46 RCTs including 4464 patients reported SCR (Supplementary Figure S5A). All TCMIs + ADs were significantly different compared with ADs, and BRE + ADs had the best P-score (0.77; Supplementary Figure S6A). Network meta-analysis results showed no significant differences among all TCMIs + ADs for SCR (Supplementary Table S5).

#### BUN

Network plots included all interventions shown that 28 RCTs including 2553 patients reported BUN (Supplementary Figure S5B). All TCMIs + ADs demonstrated significant differences compared with ADs, and STS + ADs and BRE + ADs had the better P-score (0.71 and 0.70, respectively; Supplementary Figure S6B). Network meta-analysis showed no significant differences among all TCMIs + ADs for BUN (Supplementary Table S6).

#### CCR

In total, 12 RCTs reported CCR in 928 patients randomized to receive AST + ADs, BRE + ADs, DH + ADs, GLED + ADs, and ADs alone (Supplementary Figure S5C). Only DH + ADs demonstrated a significant difference compared with ADs alone (Supplementary Figure S6C), and network meta-analysis showed no significant differences among all possible TCMIs + ADs for CCR (Supplementary Table S6).

#### β2-MG

In total, 15 RCTs reported β2-MG in 1127 patients randomized to receive AST + ADs, BRE + ADs, DH + ADs, STS + ADs, and ADs alone (Supplementary Figure S7A). Only AST + ADs demonstrated a significant difference compared with ADs alone (Supplementary Figure S8A), and network meta-analysis showed no significant differences among all possible TCMIs + ADs for β2-MG (Supplementary Table S7).

#### AEs

In total, 15 RCTs reported AEs in 1343 patients randomized to receive AST + ADs, BRE + ADs, DH + ADs, SA + ADs, and ADs alone (Supplementary Figure S7B). There were no significant differences among all interventions in terms of AEs (Supplementary Figure S8B; Supplementary Table S7). Supplementary Table S8 summarizes the detailed information including that for SA + ADs (2 RCTs, 3 AEs), DH + ADs (3 RCTs, 4 AEs), BRE + ADs (8 RCTs, 16 AEs), AST + ADs (2 RCTs, 4 AEs), and ADs alone (9 RCTs, 16 AEs). Neither GLED + ADs nor TST + ADs demonstrated any AEs in the included studies. Therefore, they may be safer than other TCMIs + ADs. Of all AEs, the incidence of cough due to ADs and BRE + ADs was the highest.

### Meta-Regression

We found evidence that multiple covariants were not the source of significant heterogeneity in most interventions (DH + ADs, GLED + ADs, SA + ADs, and STS + ADs) for primary outcomes (Supplementary Table S9). However, the results of AST + ADs in 24-h UPE and SBP were influenced by some heterogeneity (24-h UPE: ADs usage and type; SBP: ADs usage, sample size, and mean age). The sample size also affected the results of BRE + ADs for SBP.

### Publication Bias

**FIGURE 6:**
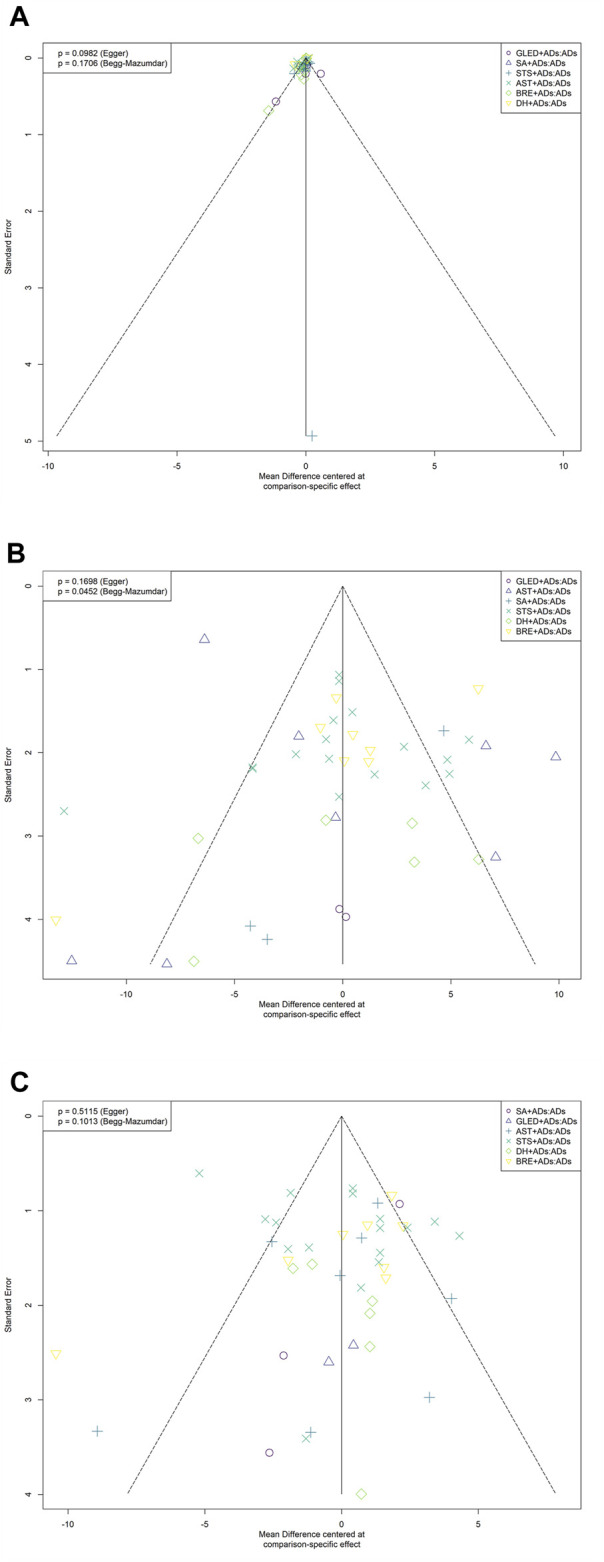
Funnel plots for primary outcomes. ADs: antihypertensive drugs; SA: salvianolate; DH: danhong; BRE: breviscapine; AST: astragalus; STS: sodium tanshinone IIA sulfonate; GLED: ginkgo leaf extract and dipyridamole. **(A)**: 24-h urinary protein excretion; **(B)**: Systolic blood pressure; **(C)**: Diastolic blood pressure.

We identified the potential publication bias for primary outcomes by using Begg’s and Egger tests, and the results of the funnel plots showed some asymmetric distributions in 24-h UPE (Egger’s: *p* = 0.0982; Begg’s: *p* = 0.1706), SBP (Egger’s: *p* = 0.1698; Begg’s: *p* = 0.0452), and DBP (Egger’s: *p* = 0.5115; Begg’s: *p* = 0.1013; Figure 6).

## Discussion

### Principal Findings

In this systematic review including 69 RCTs with 6373 patients, we comprehensively summarized and assessed the efficacy and safety of TCMIs + ADs in patients with HN. The robustness of this network meta-analysis combined with meta-regression suggested that among all TCMIs combined with ADs, SA + ADs, STS + ADs, and GLED + ADs may more efficacious in HN treatment than ADs in the primary outcomes. On the basis of the comparisons of original and sensitivity analysis results and the highest odds of possible ranking, GLED + ADs was the most efficacious intervention to improve 24-h UPE and control BP. Furthermore, BRE + ADs may be more effective in improving mALB than other interventions without GLED + ADs. No significant differences were found in the SCR, BUN, CCR, and β2-MG among the various TCMIs + ADs. Although the differences in terms of AEs between different TCMIs + ADs were nonsignificant, it is necessary to be careful regarding the application of BRE + ADs in HN patients with cough-related diseases.

Taken together, the results of this network meta-analysis have some implications for the development of TCM therapeutic interventions for clinical practice and confirmed that some TCMIs + ADs may improve HN patients’ clinical symptoms, with GLED + ADs having the most significant effects. Our result may provide certain evidence for clinical decision-making with critical assessments regarding the use of TCMIs + ADs for HN treatment.

### Relationship and Comparison with Other Studies

This network meta-analysis is more comprehensive than the previous similar meta-analysis to compare various TCMIs + ADs for HN. Previous systematic reviews have found some TCMIs + ADs to be more effective than ADs alone for HN (Sun et al., 2012; Xu et al., 2019; Li et al., 2020). However, selecting and recommending the best efficacious and safe TCMI + AD for clinical applications is warranted. We synthesized data for multiple interventions across 69 RCTs (substantially more trials than those included in other similar studies) to show the relative rankings of TCMIs + ADs for efficacy and safety. Our results somewhat provide evidence to support and create TCM guideline recommendations for HN treatment. In contrast to the observed outcomes reported in the related meta-analyses, we selected the 24-h UPE and BP as the primary outcomes to assess clinical efficacy more comprehensively and precisely according to the published data and guidelines for HN treatment. In addition, the current network meta-analysis provided the evidence of recommended TCMIs + ADs applied in HN patients at different CKD stages for clinical practice.

### Study Strengths and Limitations

We used the network meta-analysis to assess all TCMIs + ADs for HN via direct comparisons of 69 RCTs. There were no direct comparisons between the various TCMIs + ADs. Therefore, our results were unaffected by the incoherence from the direct and indirect estimates. We performed a detailed literature review and screening and conducted independent data extraction to ensure that our results comprehensive and rigorous based on identical criteria. The most stringent outcomes have been selected for the effects on HN according to the most extensive data from the published results, guidelines, and expert opinions in order to indicate the clinical value of the current study. Because different ADs (ARBs and ACEIs) are recommended to HN patients at different CKD stages, we conducted a sensitivity analysis to excluded other ADs and compared the results with the original network meta-analysis to ensure the precision of our results. The comparison results of the original network meta-analysis and sensitivity analysis demonstrated no significant changes, which further illustrated the stability and credibility of this systematic review.

There were several limitations to our study. All included studies were conducted and completed in China, which may have affected the applicability of our results. Some deficiencies in the methodological quality of included studies were follows: random sequence generation indicated high bias risk in some included studies and blinding and allocation concealment demonstrated unclear bias risk in most studies. Based on the overall poor quality of included studies, just doing such an analysis may simply hider some problems which need to be assessed, including clinical value of core outcomes, potential clinical heterogeneity, and so on. Although we conducted funnel plot, meta-regression and sensitivity analysis to explore and explain these problems. But the funnel plot results showed that the present study had potential publication bias in various interventions, which may be influenced by small sample effects in each comparison. mALB has not been comprehensively compared due to the lack of one intervention (GLED + ADs). Therefore, we could not consider mALB as the primary outcome. Although sensitivity analysis for recommended ADs at different CKD stages in patients with HN, we could not analyze the sensitivity of ARB or ACEI in each stage separately. The potential sources of heterogeneity have been detected in AST + ADs and BRE + ADs by meta-regression, possibly leading to the results of these interventions being more unstable in primary outcomes. Secondary outcomes lacked some comparisons between all TCMIs + ADs and thus could cause uncomprehensive assessment. Furthermore, the economic value may be considered when making TCMI recommendations.

### Implications for Practice

Through the synthesis of all TCMI + AD RCT evidence, this network meta-analysis provides some information for clinical physicians and health policy makers in the TCM field. When combined with ADs, SA, STS, and GLED were noted to be the best TCMIs with clinical efficacy in this network meta-analysis. SA and STS are the TCM extracts, extracted and separated from the *Salvia miltiorrhiza*, has promising efficiency for protecting CVD and alleviating kidney injury (Ma et al., 2018; Ren et al., 2019; Liang et al., 2021). SA provides anti-inflammatory, antioxidant, anti-vasodilation, and endothelial-protective activities and thus improves cardiovascular function (Fei et al., 2013; Yue et al., 2017; Ren et al., 2019). The results of the studies demonstrated that STS suppresses and protects from renal fibrosis via the expression of multiple pathways, the cardioprotective effects of which play antioxidant and anti-inflammatory roles (Gao et al., 2012; Wang et al., 2015; Cao et al., 2017). GLED can effectively prevent hypertension-induced renal injury, which was related to oxidative stress and anti-inflammatory effects (Abdel-Zaher et al., 2017; Abdel-Zaher et al., 2018). In addition, the effects of GLED may be attributed to the improvement of ACE inhibition and endothelial function (Mansour et al., 2011). Our findings confirm the efficacy of some TCMIs + ADs in improving the clinical outcomes of patients with HN. TCMIs have been approved by the National Medical Products Administration. They include TCM extracts that cause some potential risks in specific populations (Zhou and Lian 2011; Song et al., 2021). Thus, further research should focus on the clinical value of efficacy evaluation outcomes, including quality of life, number of cardiovascular events, and all-cause mortality. Moreover, TCMIs should be used with caution in clinical practice.

## Conclusion

Based on the potential benefits and safety with critical assessments, among the various TCMIs + ADs, SA + ADs, STS + ADs, and GLED + ADs may be more superior to ADs alone with the ranking and pooled effects. We noted no significant differences in safety. Considering the absence of safety evidence, TCMIs should be used with caution in clinical practice. Because of included studies with limitations in methodology, potential heterogeneity and outcomes, high-quality RCTs from the clinical standpoint of patients and professionals are warranted. Further research should focus on the clinical value of core outcomes to provide a reference value for the decision-making of clinical guidelines.

## Data Availability

The original contributions presented in the study are included in the article/Supplementary Material, further inquiries can be directed to the corresponding authors.
