# Supplementary material for: Traditional Chinese Medicine Injections Combined With Antihypertensive Drugs for Hypertensive Nephropathy: A Network Meta-Analysis
Source: Front Pharmacol. 2021 Oct 22;12:740821. doi: 10.3389/fphar.2021.740821 (PMC8570188; doi:10.3389/fphar.2021.740821)
Supplement: Supplementary file 1 [file DataSheet1.DOCX]

**Supplementary Material**

**Contents of Supplementary Figures, Tables, Files, and PRISMA checklist**

[**Figure 1 Risk of Bias Summary 3**](#_Toc83107250)

[**Figure 2 Risk of Bias of Included Studies 4**](#_Toc83107251)

[**S Figure 3 Network Plots of mAlb 5**](#_Toc83107252)

[**S Figure 4 Forest Plots and P-score of mALB 6**](#_Toc83107253)

[**S Figure 5 Network Plots of SCR, BUN and CCR 7**](#_Toc83107254)

[**S Figure 6 Forest Plots and P-score of SCR, BUN and CCR 8**](#_Toc83107255)

[**S Figure 7 Network Plots of β2-MG and Adverse events 10**](#_Toc83107256)

[**S Figure 8 Forest Plots and P-score of β2-MG and Adverse events 11**](#_Toc83107257)

[**S Table 1 Summary Table of All Included Studies 12**](#_Toc83107258)

[**S Table 2 Detailed Chemical Characterizations of Traditional Chinese Medicine Injections 18**](#_Toc83107259)

[**S Table 3 Characteristics of Included Studies 19**](#_Toc83107260)

[**S Table 4 Combined P-score of Biplots 32**](#_Toc83107261)

[**S Table 5 League table of mALB and SCR 33**](#_Toc83107262)

[**S Table 6 League table of BUN and CCR 34**](#_Toc83107263)

[**S Table 7 League table of β2-MG and Adverse events 35**](#_Toc83107264)

[**S Table 8 Adverse events 36**](#_Toc83107265)

[**S Table 9 The Meta-regression for Primary Outcomes 38**](#_Toc83107266)

[**S File 1 Protocol amendments 44**](#_Toc83107267)

[**S File 2 The Detailed Search Strategy 44**](#_Toc83107268)

[**S File 3 Reference of Included Studies 47**](#_Toc83107269)

[**S File 4 PRISMA Checklist 54**](#_Toc83107270)

# Figure 1 Risk of Bias Summary


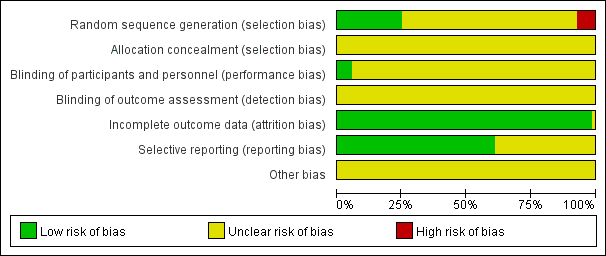


# Figure 2 Risk of Bias of Included Studies


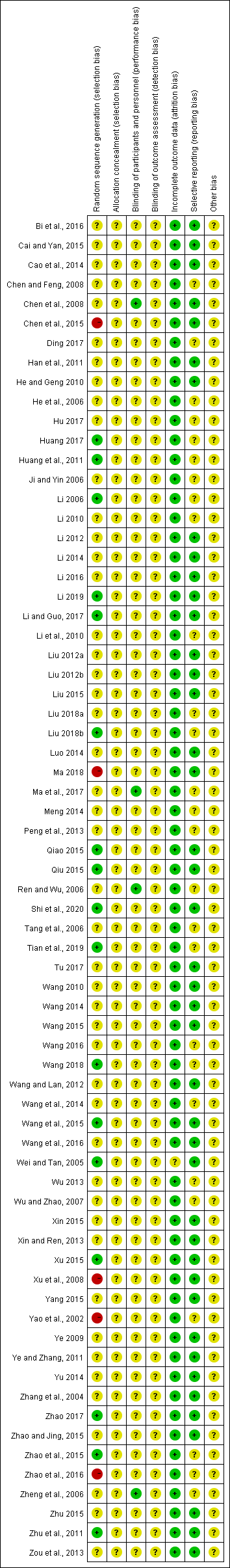


# S Figure 3 Network Plots of mAlb


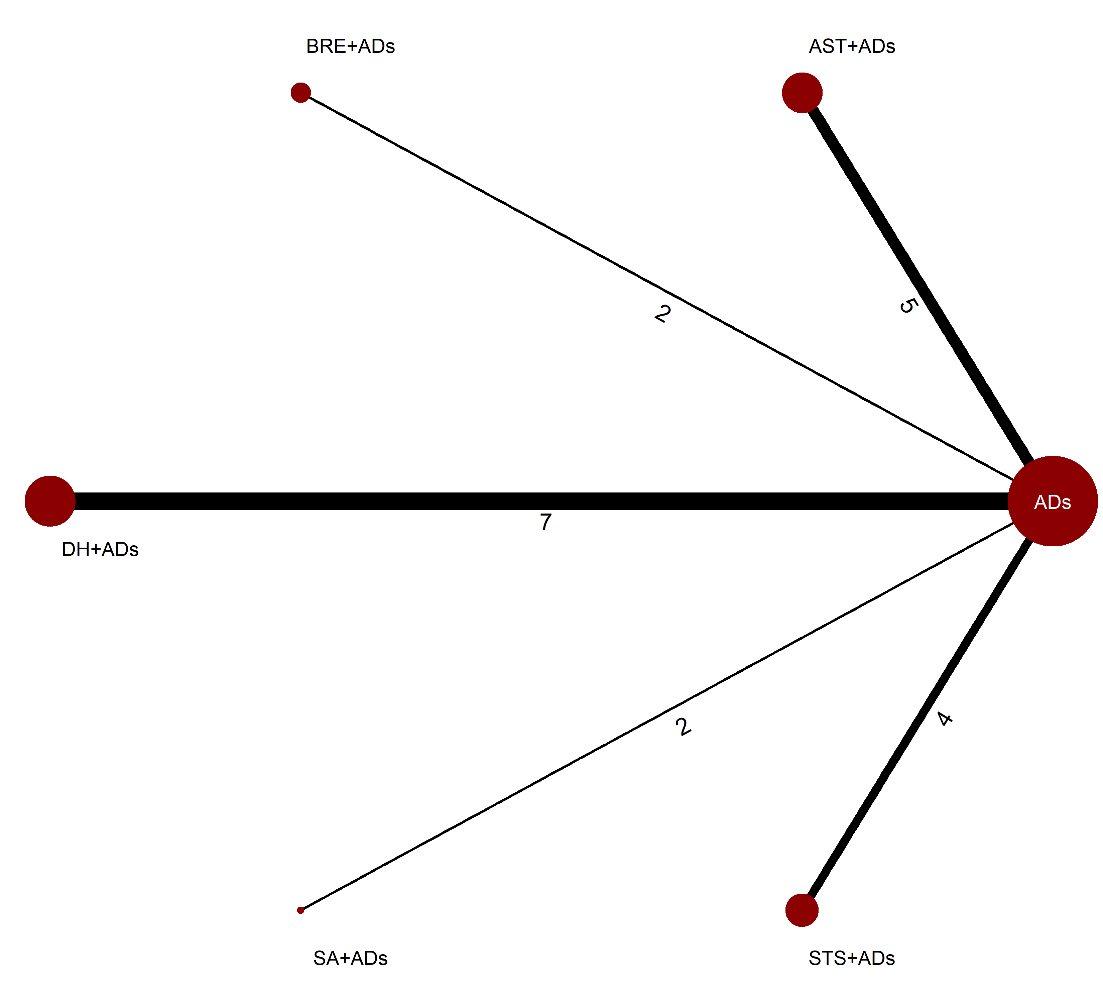


ADs: Antihypertensive drugs; SA: Salvianolate; DH: Danhong; BRE: Breviscapine; AST: Astragalus; STS: Sodium tanshinone IIA sulfonate.

mALB: micro-albuminuria.

# S Figure 4 Forest Plots and P-score of mALB


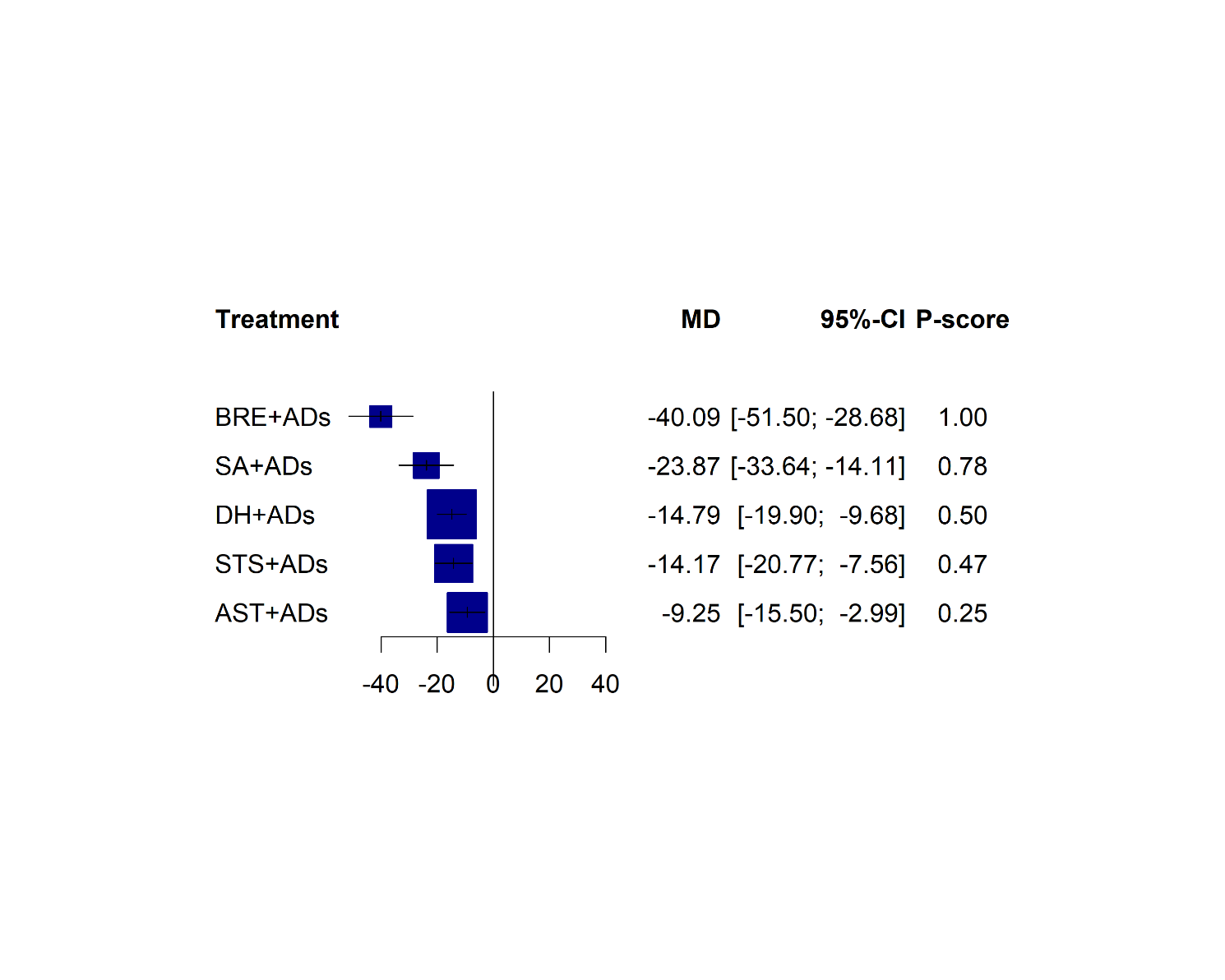


ADs: Antihypertensive drugs; SA: Salvianolate; DH: Danhong; BRE: Breviscapine; AST: Astragalus injection; STS: Sodium tanshinone IIA sulfonate; GLED.

mALB: micro-albuminuria.

# S Figure 5 Network Plots of SCR, BUN and CCR


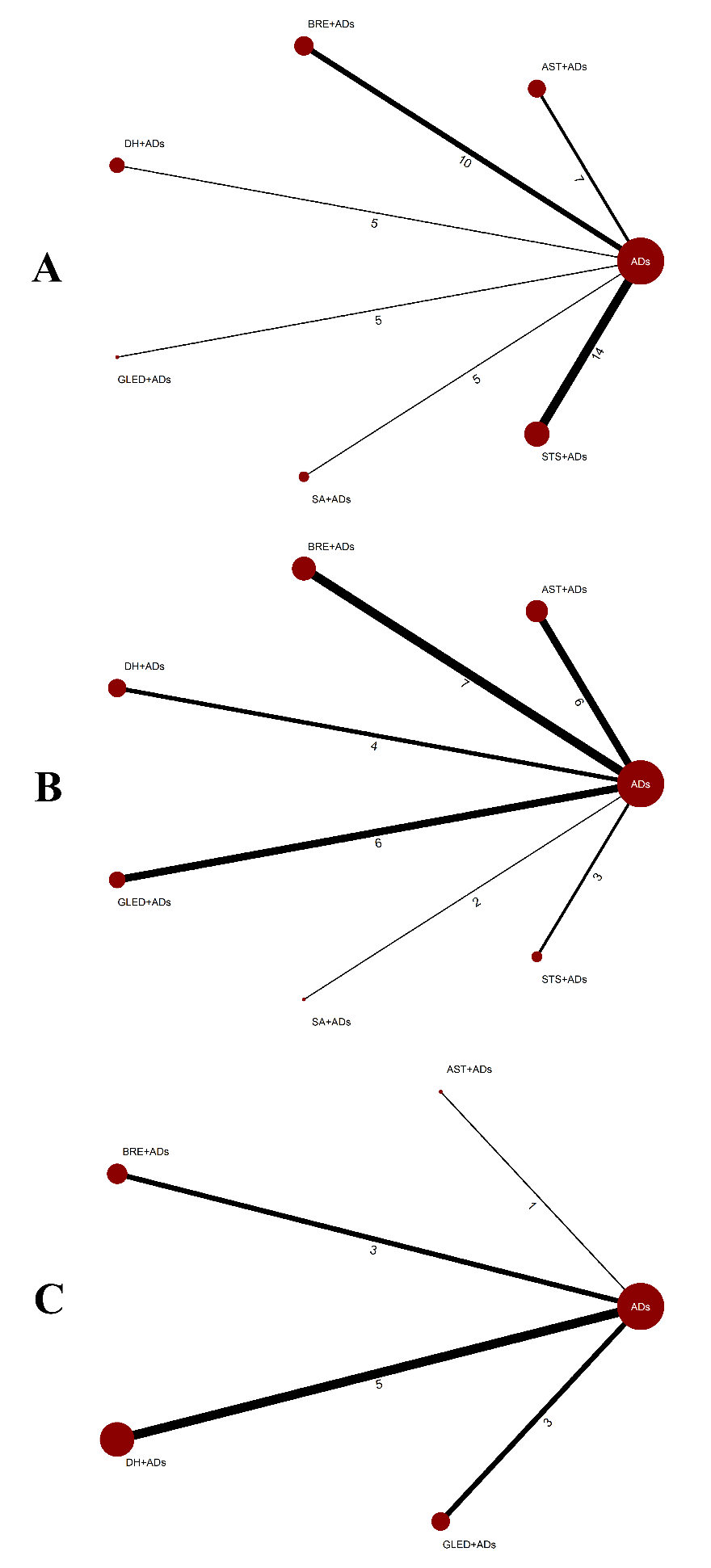


ADs: Antihypertensive drugs; SA: Salvianolate; DH: Danhong; BRE: Breviscapine; AST: Astragalus; STS: Sodium tanshinone IIA sulfonate; GLED: Ginkgo leaf extract and dipyridamole.

SCR: serum creatinine; BUN: blood urea nitrogen; CCR: creatinine clearance rate.

A: serum creatinine; B: blood urea nitrogen; C: creatinine clearance rate.

# S Figure 6 Forest Plots and P-score of SCR, BUN and CCR


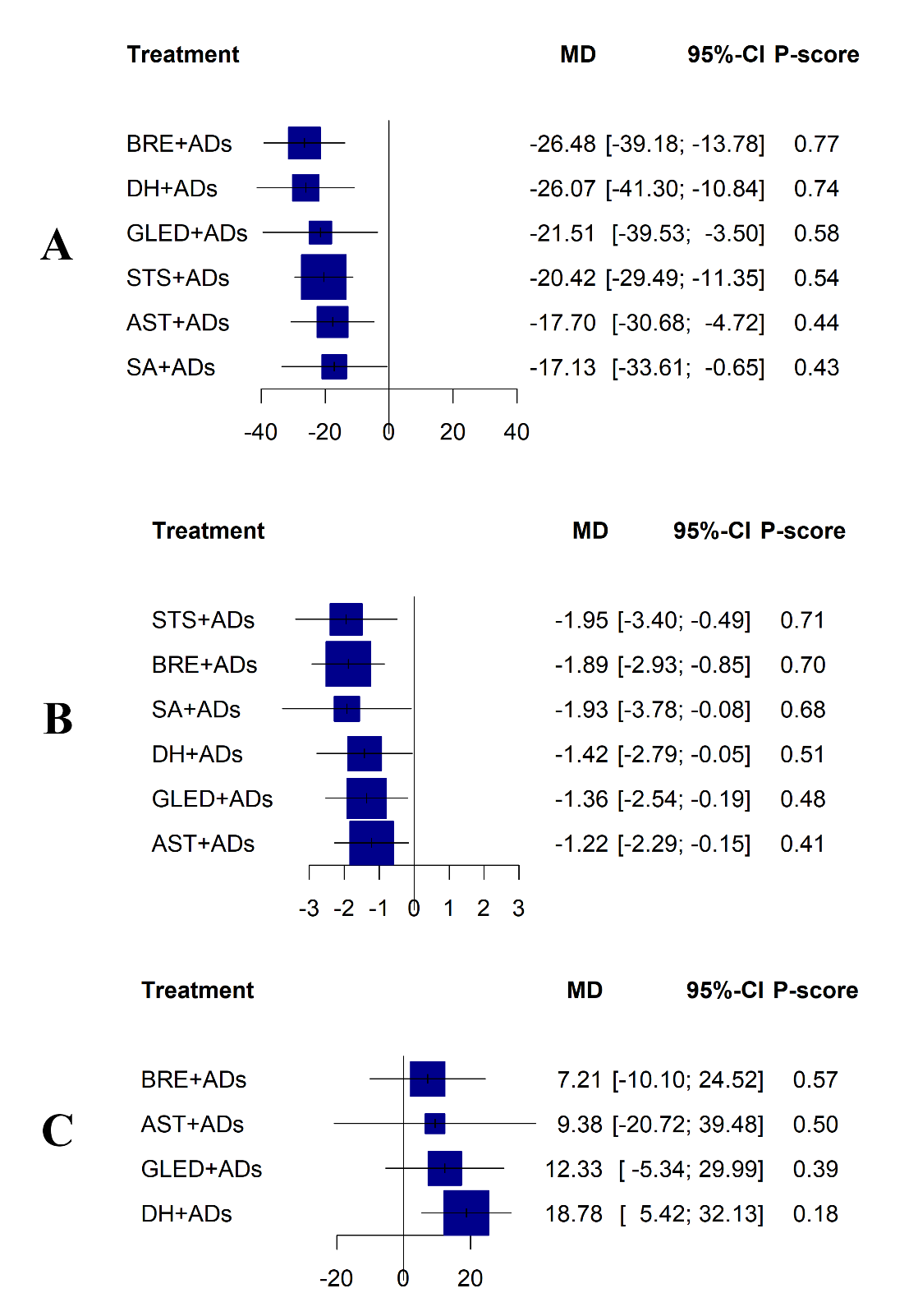


ADs: Antihypertensive drugs; SA: Salvianolate; DH: Danhong; BRE: Breviscapine; AST: Astragalus; STS: Sodium tanshinone IIA sulfonate; GLED: Ginkgo leaf extract and dipyridamole.

SCR: serum creatinine; BUN: blood urea nitrogen; CCR: creatinine clearance rate.

A: serum creatinine; B: blood urea nitrogen; C: creatinine clearance rate.

# S Figure 7 Network Plots of β2-MG and Adverse events


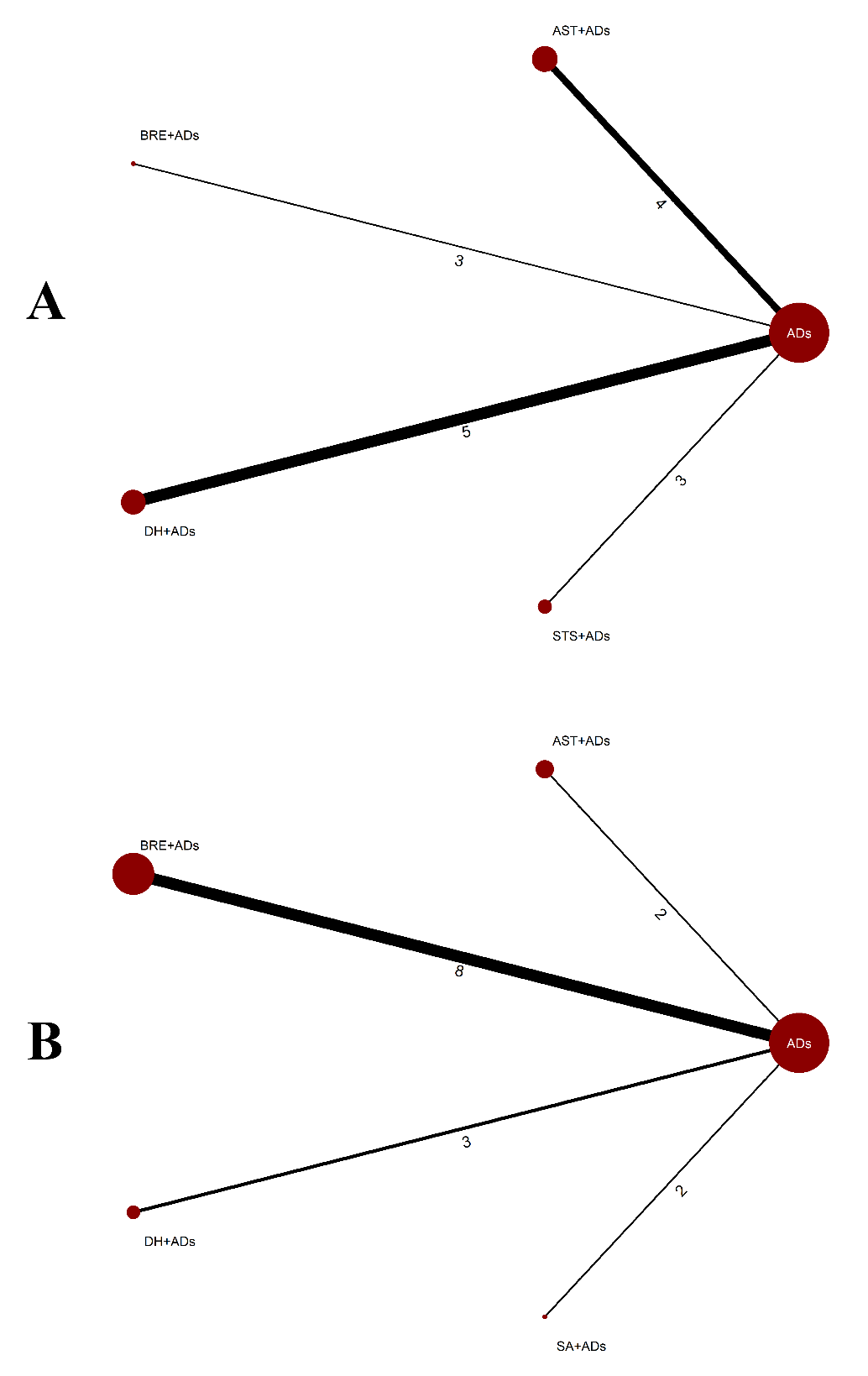


ADs: Antihypertensive drugs; SA: Salvianolate; DH: Danhong; BRE: Breviscapine; AST: Astragalus; STS: Sodium tanshinone IIA sulfonate.

β2-MG: β2-microglobulin.

A: β2-microglobulin; B: Adverse events.

# S Figure 8 Forest Plots and P-score of β2-MG and Adverse events


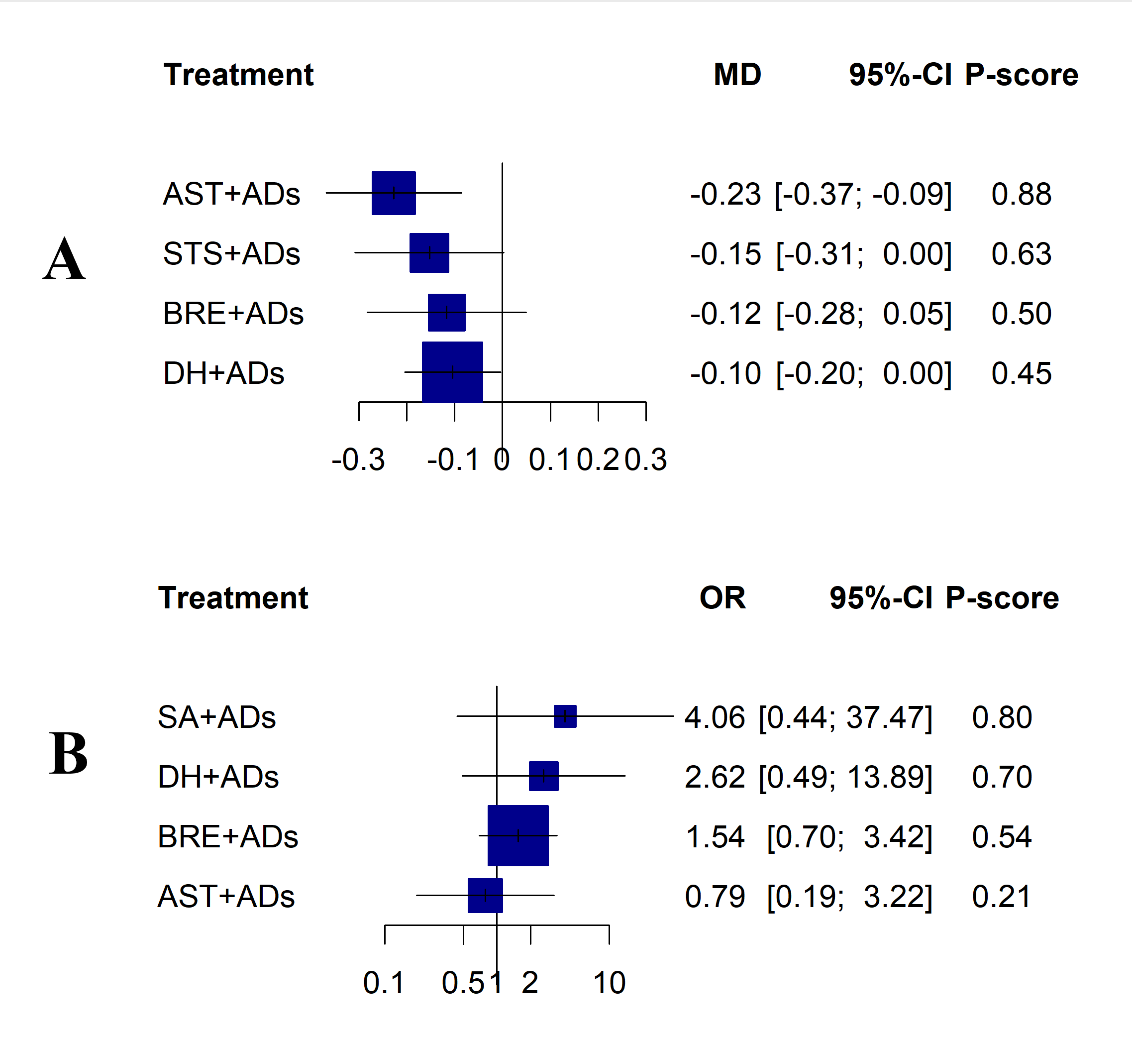


ADs: Antihypertensive drugs; SA: Salvianolate; DH: Danhong; BRE: Breviscapine; AST: Astragalus injection; STS: Sodium tanshinone IIA sulfonate.

β2-MG: β2-microglobulin.

A: β2-microglobulin; B: Adverse events.

# S Table 1 Summary Table of All Included Studies

| **Study + Publication year** | **Interventions** | | **Sample size** | | **Male** | | **Female** | | **Age (years)** | | **Course of disease (years)** | | **Detailed information of dosage** | | **Course of treatment** | **Outcomes** |
| --- | --- | --- | --- | --- | --- | --- | --- | --- | --- | --- | --- | --- | --- | --- | --- | --- |
|  | T | C | T | C | T | C | T | C | T | C | T | C | T | C |  |  |
| Ding 2017 | SA+ADs | ADs | 40 | 40 | 25 | 24 | 15 | 16 | 60.08±5.19 | 61.15±5.23 | 9.54±3.05 | 10.14±3.53 | SA (200mg/d) + ARB (80mg/d) | ARB (80mg/d) | 2 weeks | ②③④ |
| Liu 2018a | SA+ADs | ADs | 53 | 53 | 31 | 30 | 22 | 23 | 61.9±6.2 | 61.4±6.6 | 12.2±2.5 | 12.4±2.6 | SA (100mg/d) + ARB (80mg/d) | ARB (80mg/d) | 2 weeks | ④ |
| Tian et al., 2019 | SA+ADs | ADs | 45 | 45 | 20 | 21 | 25 | 24 | 45.11±6.38 | 47.31±6.12 | 5.32±0.64 | 5.44±0.31 | SA (200mg/d) + ARB (80mg/d) | ARB (80mg/d) | 12 weeks | ⑨ |
| Wang 2018 | SA+ADs | ADs | 45 | 48 | 25 | 26 | 20 | 22 | 55.89±7.65 | 55.89±7.65 | 13.92±5.92 | 13.69±5.68 | SA (200mg/d) + ARB (80mg/d) | ARB (80mg/d) | 2 weeks | ④⑤⑥ |
| Wang et al., 2015 | SA+ADs | ADs | 45 | 45 | 25 | 26 | 20 | 19 | 57±3 | 57±3 | 10±3 | 10±3 | SA (100mg/d) + ARB (80mg/d) | ARB (80mg/d) | 2 weeks | ②③④⑨ |
| Wang 2015 | SA+ADs | ADs | 45 | 45 | - | - | - | - | - | - | - | - | SA (100mg/d) + ARB (80mg/d) | ARB (80mg/d) | 2 weeks | ①②③④⑤⑥ |
| Li 2012 | DH+ADs | ADs | 42 | 42 | 24 | 23 | 18 | 19 | 65.8±14.7 | 63.4±15.2 | - | - | DH (20 ml/d) + ACEI (4 mg/d) | ACEI (4 mg/d) | 4 weeks | ①②③④⑤⑨ |
| Li et al., 2010 | DH+ADs | ADs | 102 | 102 | 70 | 75 | 32 | 27 | 68.7±6.6 | 69.7±7.8 | 8.5±2.6 | 8.9±3.2 | DH (40 ml/d) + ACEI (10mg/d) | ACEI (10mg/d) | 4 weeks | ④⑥ |
| Liu 2012a | DH+ADs | ADs | 64 | 64 | 28 | 30 | 36 | 34 | 72.5±12.8 | 73±12.5 | - | - | DH (40 ml/d) + CCB (10 mg/d), Diuretic (25 mg/d), and ARB (40 mg/d) | CCB (10 mg/d), Diuretic (25 mg/d), and ARB (40 mg/d) | 1 weeks | ②③④⑤⑧ |
| Meng 2014 | DH+ADs | ADs | 32 | 32 | 42 | | 22 | | 58.3±11.7 | 58.3±11.7 | 7.6±3.8 | 7.6±3.8 | DH (40 ml/d) + CCB (5-10mg) | CCB (5-10mg) | 4 weeks | ⑥⑦⑨ |
| Peng et al., 2013 | DH+ADs | ADs | 31 | 34 | - | - | - | - | 63.8±9.4 | 63.8±9.4 | 6.8±7.3 | 6.8±7.3 | DH (40 ml/d) + CCB (5-10mg) | CCB (5-10mg) | 4 weeks | ⑥⑦⑨ |
| Wang 2014 | DH+ADs | ADs | 40 | 40 | 20 | 19 | 20 | 21 | 68.1±7.9 | 67.8±7.1 | 2.6±0.9 | 1.9±2.1 | DH (40 ml/d) + ARB (150 mg/d) | ARB (150 mg/d) | 6 weeks | ②③⑥⑦⑧ |
| Wang 2010 | DH+ADs | ADs | 80 | 60 | 59 | 41 | 26 | 19 | 77±6.45 | 76.2±6.75 | - | - | DH (40 ml/d) + CCB (10 mg/d), Diuretic (25 mg/d), and ARB (40-80 mg/L) | CCB (10 mg/d), Diuretic (25 mg/d), and ARB (40-80 mg/L) | 4 weeks | ②③④⑤⑧ |
| Wang 2016 | DH+ADs | ADs | 40 | 40 | 24 | 22 | 16 | 18 | 63.4±9.6 | 62.6±9.1 | 12.7±5.9 | 13.2±5.7 | DH (40 ml/d) + ACEI | ACEI | 4 weeks | ①④⑤⑥ |
| Xin 2015 | DH+ADs | ADs | 34 | 34 | 45 | | 23 | | 65.4±2.3 | 65.4±2.3 | 2.5±1.1 | 2.5±1.1 | DH (40 ml/d) + ARB (150 mg/d) | ARB (150 mg/d) | 4 weeks | ②③⑥⑦⑧ |
| Xin and Ren, 2013 | DH+ADs | ADs | 30 | 30 | 40 | | 20 | | 73.22±1.05 | 73.22±1.05 | - | - | DH (40 ml/d) + ARB (150 mg/d) | ARB (150 mg/d) | 6 weeks | ②③⑥⑦⑧ |
| Chen et al., 2008 | BRE+ADs | ADs | 28 | 28 | 19 | 20 | 9 | 8 | 45.6±20.3 | 46.4±21.1 | 10.2±5.1 | 11.3±5.3 | BRE (20 mg/d) + ACEI | ACEI | 4 weeks | ①④⑤⑧⑨ |
| He and Geng 2010 | BRE+ADs | ADs | 84 | 84 | 49 | 48 | 35 | 36 | 69±11 | 68±11 | 10.9±8.5 | 10.2±9.1 | BRE (20 mg/d) +ACEI (12.5-50 mg,tid) | ACEI (12.5-50 mg,tid) | 4 weeks | ②③④⑤⑥⑨ |
| Liu 2012b | BRE+ADs | ADs | 26 | 24 | 12 | 10 | 14 | 14 | 55±4 | 53±5 | 9.1±3.4 | 8.5±2.6 | BRE (40 mg/d) + ACEI (10mg,tid) | ACEI (10mg,tid) | 2 weeks | ②③④⑥ |
| Ma 2018 | BRE+ADs | ADs | 33 | 33 | 19 | 20 | 14 | 13 | 48±3.14 | 48.99±2.98 | 15.77±0.67 | 14.95±0.84 | BRE (20 mg/d) + ARB (20 mg/d) | ARB (20 mg/d) | 4 weeks | ②③④⑦⑧ |
| Qiao 2015 | BRE+ADs | ADs | 79 | 79 | 98 | | 60 | | 48.01±3.15 | 48.01±3.15 | 15.78±0.68 | 15.78±0.68 | BRE (20 mg/d) + ACEI (25-75 mg/d) and CCB (10-60 mg/d) | ACEI (25-75 mg/d) and CCB (10-60 mg/d) | 4 weeks | ①②③④⑤ |
| Ren and Wu, 2006 | BRE+ADs | ADs | 30 | 30 | 21 | 22 | 9 | 8 | 59.5±7.6 | 58.5±7.6 | - | - | BRE (30 mg/d) + ACEI (25-75 mg/d) and CCB (20-60 mg/d) | ACEI (25-75 mg/d) and CCB (20-60 mg/d) | 4 weeks | ①④⑤⑨ |
| Wang and Lan, 2012 | BRE+ADs | ADs | 52 | 51 | 56 | | 47 | | 62.4±4.8 | 62.4±4.8 | 8.3±5.4 | 8.3±5.4 | BRE (25 mg/d) + ACEI (20 mg/d) | ACEI (20 mg/d) | 4 weeks | ①②③④⑤⑨ |
| Wei and Tan, 2005 | BRE+ADs | ADs | 40（36） | 36 | 30 | 28 | 10 | 8 | 60.25±9.74 | 64.15±9 | 17.2 | 17.7 | BRE (50 mg/d) + amlodipine (5 mg/d), captopril (75 mg/d), urapidil (60-90 mg/d) | amlodipine (5 mg/d), captopril (75 mg/d), urapidil (60-90 mg/d) | 4 weeks | ①④⑤⑦⑨ |
| Ye 2009 | BRE+ADs | ADs | 40 | 35 | 24 | 20 | 16 | 15 | 47.3±18.2 | 46.9±16.1 | 18.3±7.7 | 11.5±1.3 | BRE (20 mg/d) +ARB (100 mg/d) | ARB (100 mg/d) | 4 weeks | ②③④⑦⑧⑨ |
| Zhang et al., 2004 | BRE+ADs | ADs | 47 | 47 | 53 | | 41 | | 60±8 | - | - | - | BRE (100 mg/d) + ACEI (5-10 mg/d) | ACEI (5-10 mg/d) | 4 weeks | ①②③⑨ |
| Zhao et al., 2016 | BRE+ADs | ADs | 40 | 40 | 50 | | 30 | | 52.5±7.1 | 52.5±7.1 | 8.1±1.5 | 8.1±1.5 | BRE (20 mg/d) + ACEI (5 mg/d) | ACEI (5 mg/d) | 4 weeks | ②③ |
| Zheng et al., 2006 | BRE+ADs | ADs | 37 | 35 | 23 | 22 | 14 | 13 | 57.25±6.82 | 56.5±6.89 | - | - | BRE (30 ml/d) + ACEI (25-75 mg/d) and CCB (10-60 mg/d) | ACEI (25-75 mg/d) and CCB (10-60 mg/d) | 4 weeks | ①④⑤⑨ |
| Chen et al., 2015 | AST+ADs | ADs | 90 | 90 | 54 | 51 | 36 | 39 | 43.25±9.61 | 42.67±9.72 | 5.47±2.02 | 5.63±2.15 | AST (30 ml/d) + combination of multiple ADs | combination of multiple ADs | 3 weeks | ①②③④ |
| Han et al., 2011 | AST+ADs | ADs | 40 | 38 | 26 | 22 | 14 | 16 | 57.2±3.5 | 57.8±3.3 | 13.5±3.6 | 12.7±2.1 | AST (40 ml/d) + ARB (40 mg/d) and ARB (150 mg/d) | ARB (40 mg/d) and ARB (150 mg/d) | 4 weeks | ①②③④⑤ |
| Huang 2017 | AST+ADs | ADs | 63 | 63 | 41 | 40 | 22 | 23 | 58.36±6.87 | 57.93±6.97 | 5.84±1.16 | 5.62±1.38 | AST (30 ml/d) +ARB (150-300 mg/d) | ARB (150-300 mg/d) | 4 weeks | ①⑥⑨ |
| Huang et al., 2011 | AST+ADs | ADs | 45 | 45 | 54 | | 36 | | 62.25±8.52 | 62.25±8.52 | - | - | AST (30 ml/d) + ARB (150 mg/d) | ARB (150 mg/d) | 4 weeks | ⑥⑦ |
| Ji and Yin 2006 | AST+ADs | ADs | 54 | 40 | 36 | 23 | 18 | 17 | 56.3 | 54.6 | 7.8 | 7.4 | AST (20 ml/d) + combination of multiple ADs | combination of multiple ADs | 4 weeks | ⑥⑦ |
| Li 2006 | AST+ADs | ADs | 30 | 30 | 18 | 17 | 12 | 13 | 70.63±3.8 | 69.97±3.73 | 11.91±5.4 | 12.31±5.98 | AST (100 ml/d) + ACEI (12.5 mg/d) | ACEI (12.5 mg/d) | 8 weeks | ⑥⑦ |
| Luo 2014 | AST+ADs | ADs | 62 | 62 | 36 | 46 | 26 | 16 | 45.1±4.9 | 46.3±4.5 | 10.63±4.03 | 10.63±4.03 | AST (10-20 ml/d) + ARB (80 mg/d) and CCB (2.5 mg/d) | ARB (80 mg/d) and CCB (2.5 mg/d) | 8 weeks | ①②③④⑤⑨ |
| Tang et al., 2006 | AST+ADs | ADs | 30 | 30 | 18 | 16 | 12 | 14 | 67.1±9.94 | 62.96±9.54 | - | - | AST (60 ml/d) + ACEI and CCB | ACEI and CCB | 3.5 weeks | ① |
| Wu and Zhao, 2007 | AST+ADs | ADs | 23 | 23 | 15 | 16 | 8 | 7 | 54±8 | 55±9 | - | - | AST (50 ml/d) + combination of multiple ADs | combination of multiple ADs | 4 weeks | ① |
| Xu et al., 2008 | AST+ADs | ADs | 26 | 22 | 28 | | 20 | | 63.5±14.1 | 63.5±14.1 | - | - | AST (20 ml/d) +ARB (80 mg /d) and CCB (5 mg/d) | ARB (80 mg /d) and CCB (5 mg/d) | 3 weeks | ①②③⑥ |
| Xu 2015 | AST+ADs | ADs | 40 | 40 | 23 | 21 | 17 | 19 | 58.71±5.19 | 58.34±5.56 | 12.42±6.23 | 12.31±6.12 | AST (50 ml/d) + ARB (150 mg/d) | ARB (150 mg/d) | 4 weeks | ②③④⑤⑧ |
| Yang 2015 | AST+ADs | ADs | 80 | 78 | 51 | 50 | 29 | 28 | - | - | - | - | AST (40 ml/d) + ARB (50mg) and Diuretic (12.5mg) | ARB (50mg) and Diuretic (12.5mg) | 4 weeks | ①②③ |
| Zhao 2017 | AST+ADs | ADs | 66 | 66 | 76 | | 46 | | 57.33±10.29 | 57.33±10.29 | 7.62±1.03 | 7.62±1.03 | AST (20 ml/d) + ARB (150 mg/d) | ARB (150 mg/d) | 4 weeks | ①②③④⑤ |
| Zhao and Jing, 2015 | AST+ADs | ADs | 45 | 45 | 23 | 22 | 22 | 23 | 67.6±8.2 | 68.4±8.7 | 10.1±4.3 | 10.8±4.4 | AST (40 ml/d) + ACEI and CCB | ACEI and CCB | 4 weeks | ①②③④⑤⑦ |
| Zhao et al., 2015 | AST+ADs | ADs | 56 | 56 | 31 | 32 | 25 | 24 | 62.1±7.9 | 61.6±8.2 | 7.5±3.3 | 7.2±3.1 | AST (30 ml/d) + ARB (150-300 mg/d) | ARB (150-300 mg/d) | 4 weeks | ①④⑤ |
| Yao et al., 2002 | AST+ADs | ADs | 32 | 32 | 20 | 18 | 12 | 12 | 65.1±12.9 | 64.2±13.3 | 15.6±6.8 | 14.8±7.5 | AST (40 ml/d) + ACEI (10 mg) and CCB (5 mg) | ACEI (10 mg) and CCB (5 mg) | 3 weeks | ① |
| Bi et al., 2016 | STS+ADs | ADs | 50 | 50 | - | - | - | - | 53.51±8.64 | 54.51±9.31 | 11.3±5.1 | 12.6±6.1 | STS (50 mg/d) + ARB (150 mg/d) | ARB (150 mg/d) | 4 weeks | ①②③ |
| Cao et al., 2014 | STS+ADs | ADs | 45 | 44 | 23 | 21 | 22 | 23 | 63.1±16.8 | 62.3±17.6 | 12.4±6.2 | 10.2±8 | STS (60 mg/d) + ARB (150 mg/d) | ARB (150 mg/d) | 4 weeks | ①②③⑥⑦ |
| Hu 2017 | STS+ADs | ADs | 40 | 40 | 24 | 22 | 16 | 18 | 58.64±4.39 | 59.47±3.54 | 12.63±4.78 | 13.54±3.38 | STS (40 mg/d) + ARB (80 mg/d) | ARB (80 mg/d) | 2 weeks | ④⑤⑥ |
| Li 2019 | STS+ADs | ADs | 30 | 30 | 14 | 13 | 16 | 17 | 51.67±2.49 | 51.69±2.51 | 7.69±1.57 | 7.65±1.58 | STS (60 mg/d) + ARB (50 mg/d) | ARB (50 mg/d) | 2 weeks | ①②③④⑦ |
| Li and Guo, 2017 | STS+ADs | ADs | 100 | 100 | 53 | 51 | 47 | 49 | 63.5±8 | 64.1±8.1 | 5.1±1.8 | 5.8±1.9 | STS (60 mg/d) + ARB (200 mg/d) | ARB (200 mg/d) | 2 weeks | ①②③④ |
| Li 2016 | STS+ADs | ADs | 30 | 30 | 20 | 15 | 10 | 15 | 35.83±15.33 | 36.85±16.33 | - | - | STS (60 mg/d) + ARB (80 mg/d) | ARB (80 mg/d) | 4 weeks | ②③④ |
| Li 2014 | STS+ADs | ADs | 115 | 115 | 123 | | 107 | | 62.4±7.9 | 62.4±7.9 | 5.2±1.9 | 5.2±1.9 | STS (60 mg/d) + ARB (80 mg/d) | ARB (80 mg/d) | 2 weeks | ①②③④ |
| Liu 2015 | STS+ADs | ADs | 45 | 45 | 25 | 28 | 20 | 17 | 51.8±3.9 | 52.1±4.2 | 8.2±2.9 | 8±3.2 | STS (20 mg/d) + ARB (50 mg/d) | ARB (50 mg/d) | 2 weeks | ①②③④ |
| Liu 2018b | STS+ADs | ADs | 34 | 34 | 19 | 18 | 15 | 16 | 59.2±11.3 | 59.6±11.2 | 9.8±1.2 | 9.9±1.3 | STS (60 mg/d) + ARB (80 mg/d) | ARB (80 mg/d) | 2 weeks | ②③ |
| Ma et al., 2017 | STS+ADs | ADs | 36 | 36 | 21 | 20 | 15 | 16 | 57.5±5.91 | 56.5±6.86 | 18±4.26 | 16±3.78 | STS (60 mg/d) + ARB (80 mg/d) | ARB (80 mg/d) | 2 weeks | ④⑤⑥ |
| Qiu 2015 | STS+ADs | ADs | 54 | 54 | 62 | | 46 | | 62.5±7.8 | 62.5±7.8 | 11.3±5.2 | 11.3±5.2 | STS (60 mg/d) + ARB (80 mg/d) | ARB (80 mg/d) | 2 weeks | ①②③④ |
| Shi et al., 2020 | STS+ADs | ADs | 47 | 47 | 29 | 28 | 18 | 19 | 59.17±10.85 | 58.92±10.71 | 11.32±3.57 | 11.24±3.42 | STS (50 mg/d) + ARB (150 mg/d) | ARB (150 mg/d) | 8 weeks | ②③④⑤⑥⑦ |
| Tu 2017 | STS+ADs | ADs | 64 | 64 | 68 | | 60 | | 59.5±9.2 | 59.5±9.2 | 12.5±4.07 | 12.5±4.07 | STS (60 mg/d) + ARB (80 mg/d) | ARB (80 mg/d) | 2 weeks | ①②③④ |
| Wang et al., 2016 | STS+ADs | ADs | 65 | 65 | 34 | 35 | 31 | 30 | 58.4±2.9 | 57.4±3.2 | 6.3±1.4 | 6.4±1.2 | STS (60 mg/d) + ARB (80 mg/d) | ARB (80 mg/d) | 2 weeks | ①②③ |
| Wu 2013 | STS+ADs | ADs | 25 | 25 | 27 | | 23 | | 43.4±7.3 | 43.4±7.3 | 7.6±1.4 | 7.6±1.4 | STS (40 mg/d) + ARB (50 mg/d) | ARB (50 mg/d) | 2 weeks | ①④ |
| Yu 2014 | STS+ADs | ADs | 34 | 33 | 21 | 24 | 13 | 9 | 62.7±10.5 | 64.6±11.1 | 9.5±2.63 | 10.5±2.65 | STS (60 mg/d) + ARB (80 mg/d) | ARB (80 mg/d) | 2 weeks | ①②③④ |
| Zhu et al., 2011 | STS+ADs | ADs | 30 | 30 | 15 | 16 | 15 | 14 | 54.8±7.7 | 53.1±8.9 | 7.07±1.73 | 7.71±12.9 | STS (40 mg/d) + ARB (50 mg/d) | ARB (50 mg/d) | 2 weeks | ①②③④ |
| Zhu 2015 | STS+ADs | ADs | 49 | 49 | 27 | 28 | 22 | 21 | 58.6±7.2 | 60.4±5.9 | 5±1.34 | 3±0.89 | STS (60 mg/d) + ARB (80 mg/d) | ARB (80 mg/d) | 2 weeks | ①②③ |
| Zou et al., 2013 | STS+ADs | ADs | 40 | 40 | 23 | 22 | 17 | 18 | 62±10 | 63±8 | 10±8 | 10±6 | STS (60 mg/d) + ARB (80 mg/d) | ARB (80 mg/d) | 2 weeks | ①②③④ |
| Cai and Yan, 2015 | GLED+ADs | ADs | 30 | 30 | 16 | 15 | 14 | 15 | 72.5±3.68 | 72.2±3.43 | - | - | GLED (20 ml/d) + ACEI (10 mg/d) | ACEI (10 mg/d) | 4 weeks | ①②③④⑤⑧ |
| Chen and Feng, 2008 | GLED+ADs | ADs | 35 | 33 | 24 | 23 | 11 | 10 | 59.5±7.37 | 58.5±7.45 | - | - | GLED (20 ml/d) + ARB (80-160 mg/d) | ARB (80-160 mg/d) | 4 weeks | ①④⑤ |
| He et al., 2006 | GLED+ADs | ADs | 18 | 19 | - | - | - | - | - | - | - | - | GLED (20 ml/d) + ACEI (10 mg/d) | ACEI (10 mg/d) | 4 weeks | ①⑤⑧ |
| Li 2010 | GLED+ADs | ADs | 45 | 45 | 22 | 23 | 23 | 22 | 64.2±10.1 | 63.8±9.7 | - | - | GLED (20 ml/d) + CCB (2.5-10 mg/d) | CCB (2.5-10 mg/d) | 4 weeks | ④⑤ |
| Wang et al., 2014 | GLED+ADs | ADs | 40 | 38 | 23 | 22 | 17 | 16 | 69.1±3.5 | 66.1±4.1 | 15.8±4.8 | 16.1±5.3 | GLED (30 ml/d) + combination of multiple ADs | combination of multiple ADs | 3 weeks | ①④⑤⑧ |
| Ye and Zhang, 2011 | GLED+ADs | ADs | 27 | 27 | 32 | | 22 | | 75.7±4.1 | 75.7±4.1 | - | - | GLED (20 ml/d) + ACEI (10 mg/d) | ACEI (10 mg/d) | 4 weeks | ①②③④⑤ |

Groups: T: treatment group; C: control group.

Interventions：SA: Salvianolate injection; DH: Danhong injection; BRE: Breviscapine injection; AST: Astragalus injection; STS: Sodium tanshinone IIA sulfonate injection; GLED: Ginkgo Leaf Extract and Dipyridamole injection; ADs: Antihypertensive Drugs.

Detail types of ADs：ARB: Angiotensin Receptor Blocker; ACEI: Angiotensin converting enzyme inhibitor; CCB: Calcium Channel Blockers; Diuretic.

Outcomes: ①24-hour urinary protein excretion (24-hour UPE); ②systolic blood pressure (SBP); ③diastolic blood pressure (DBP); ④serum creatinine (SCR); ⑤blood urea nitrogen (BUN); ⑥micro-albuminuria (mALB); ⑦β2-microglobulin (β2-MG); ⑧creatinine clearance rate (CCR); ⑨adverse events (AEs).

# S Table 2 Detailed Chemical Characterizations of Traditional Chinese Medicine Injections

| **Traditional Chinese medicine Injections*** | **Main chemical characterization** | **Molecular Formula** | **Median of test range (mg/ml)** |
| --- | --- | --- | --- |
| **Salvianolate injection** | Monomethyl lithospermate B | C36H28O16-2.Mg+2 | 0.234 |
| **Danhong injection** | Salvianic acid A | C9H10O5 | 1.357 |
|  | Salvianolic acid B | C36H30O16 | 0.231 |
|  | 3,4-Dihydroxybenzaldehyde | C7H6O3 | 0.213 |
|  | Rosmarinic acid | C18H16O8 | 0.208 |
|  | Salvianolic acid A | C26H22O10 | 0.187 |
|  | Salvianolic acid D | C20H18O10 | 0.14 |
|  | Lithospermic acid | C27H22O12 | 0.085 |
| **Breviscapine injection** | Scutellarin | C21H18O12 | 0.489 |
|  | Dicaffeoylquinic acids | C25H24O12 | 0.666 |
|  | Caffeic acid | C9H8O4 | 0.098 |
|  | Apigenin-7-O-glucronide | C21H18O11 | 0.089 |
|  | Chlorogenic acid | C16H18O9 | 0.038 |
| **Astragalus injection** | Astragaloside | C14H68O14 | 0.3885 |
| **Sodium tanshinone IIA sulfonate injection** | Sodium tanshinone IIA sulfonate | C19H17O3·SO3Na | 0.14 |
| **Ginkgo Leaf Extract and Dipyridamole injection** | Total flavonel flavonoids | C15H10O6; C15H10O7; C16H12O7 | 0.2 |
|  | Dipyridamole | C24H40N8O4 | 0.08 |

***Reference**

Chen, W.,Wei, W.,Zhao, P. and Jin, F. Y. (2015). Study on Fingerprint of Main Components of Yinxingdamo Injection. Journal of GuiZhou University of Traditional Chinese Medicine. 37, 13-21

Du, J. J.,Qu, X. L. and Zhang, Q. (2019). Determination of Components in Danhong Injection by HPLC. World Chinese Medicine. 14, 1373-1377

Liu, G. L.,Xu, W. L. and Wang, Z. (2017). Simultaneous determination of ten constituents in Dengzhan Xixin Injection by HPLC. Chinese Traditional Patent Medicine. 39, 2521-2524

Shi, R. T.,Zhang, Q. W.,Yao, H. and Ye, X. H. (2016). Determination of Salvia Miltiorrhiza Magnesium Acetate in Salvia Miltiorrhiza Polyphenolate for Injection by HPLC. Anhui Medical and Pharmaceutical Journal. 20, 466-468

Xiang, H. X. and Wang, S. J. (2009). Determination of Sodium Tanshinone Ⅱ A Sulfonate for injection by RP-HPLC. Coal and Chemical Industry. 32, 56-57+77

Yu, D. S.,Wei, D.,Yao, C. and Pan, L. Q. (2007). Determination of astragaloside IV in microemulsion of Astragalus injection concentrate by HPLC-ELSD. Journal of Anhui University of Chinese Medicine. 26, 36-38

# S Table 3 Characteristics of Included Studies

| Study | Formulation | Source* | Raw material  (traditional Chinese medicine) | Quality control reported? (Y/N) | Chemical analysis reported? (Y/N) |
| --- | --- | --- | --- | --- | --- |
| Ding 2017 | Salvianolate injection | Shanghai Lvgu Shengmingyuan Medicine Co., Ltd. (SFDA approval number: Z20050246) | Salvia miltiorrhiza Bunge [Lamiaceae] | Y - Prepared according to People's Republic of China Pharmacopoeia | Y-HPLC |
| Liu 2018a | Salvianolate injection | Shanghai Lvgu Shengmingyuan Medicine Co., Ltd. (SFDA approval number: Z20050246) | Salvia miltiorrhiza Bunge [Lamiaceae] | Y - Prepared according to People's Republic of China Pharmacopoeia | Y-HPLC |
| Tian et al., 2019 | Salvianolate injection | Shanghai Lvgu Shengmingyuan Medicine Co., Ltd. (SFDA approval number: Z20050246) | Salvia miltiorrhiza Bunge [Lamiaceae] | Y - Prepared according to People's Republic of China Pharmacopoeia | Y-HPLC |
| Wang 2018 | Salvianolate injection | Shanghai Lvgu Shengmingyuan Medicine Co., Ltd. (SFDA approval number: Z20050246) | Salvia miltiorrhiza Bunge [Lamiaceae] | Y - Prepared according to People's Republic of China Pharmacopoeia | Y-HPLC |
| Wang et al., 2015 | Salvianolate injection | Shanghai Lvgu Shengmingyuan Medicine Co., Ltd. (SFDA approval number: Z20050246) | Salvia miltiorrhiza Bunge [Lamiaceae] | Y - Prepared according to People's Republic of China Pharmacopoeia | Y-HPLC |
| Wang 2015 | Salvianolate injection | Shanghai Lvgu Shengmingyuan Medicine Co., Ltd. (SFDA approval number: Z20050246) | Salvia miltiorrhiza Bunge [Lamiaceae] | Y - Prepared according to People's Republic of China Pharmacopoeia | Y-HPLC |
| Li 2012 | Danhong injection | Shandong Danhong Pharmaceutical Co., Ltd. (SHANDONG BUCHANG PHARMACEUTICALS CO., LTD.) (SFDA approval number: Z20026866) | Salvia miltiorrhiza Bunge [Lamiaceae];  Carthamus tinctorius L. [Asteraceae] | Y - Prepared according to People's Republic of China Pharmacopoeia | Y-HPLC |
| Li et al., 2010 | Danhong injection | Shandong Danhong Pharmaceutical Co., Ltd. (SHANDONG BUCHANG PHARMACEUTICALS CO., LTD.) (SFDA approval number: Z20026866) | Salvia miltiorrhiza Bunge [Lamiaceae];  Carthamus tinctorius L. [Asteraceae] | Y - Prepared according to People's Republic of China Pharmacopoeia | Y-HPLC |
| Liu 2012a | Danhong injection | Shandong Danhong Pharmaceutical Co., Ltd. (SHANDONG BUCHANG PHARMACEUTICALS CO., LTD.) (SFDA approval number: Z20026866) | Salvia miltiorrhiza Bunge [Lamiaceae];  Carthamus tinctorius L. [Asteraceae] | Y - Prepared according to People's Republic of China Pharmacopoeia | Y-HPLC |
| Meng 2014 | Danhong injection | Shandong Danhong Pharmaceutical Co., Ltd. (SHANDONG BUCHANG PHARMACEUTICALS CO., LTD.) (SFDA approval number: Z20026866) | Salvia miltiorrhiza Bunge [Lamiaceae];  Carthamus tinctorius L. [Asteraceae] | Y - Prepared according to People's Republic of China Pharmacopoeia | Y-HPLC |
| Peng et al., 2013 | Danhong injection | Shandong Danhong Pharmaceutical Co., Ltd. (SHANDONG BUCHANG PHARMACEUTICALS CO., LTD.) (SFDA approval number: Z20026866) | Salvia miltiorrhiza Bunge [Lamiaceae];  Carthamus tinctorius L. [Asteraceae] | Y - Prepared according to People's Republic of China Pharmacopoeia | Y-HPLC |
| Wang 2014 | Danhong injection | Shandong Danhong Pharmaceutical Co., Ltd. (SHANDONG BUCHANG PHARMACEUTICALS CO., LTD.) (SFDA approval number: Z20026866) | Salvia miltiorrhiza Bunge [Lamiaceae];  Carthamus tinctorius L. [Asteraceae] | Y - Prepared according to People's Republic of China Pharmacopoeia | Y-HPLC |
| Wang 2010 | Danhong injection | Shandong Danhong Pharmaceutical Co., Ltd. (SHANDONG BUCHANG PHARMACEUTICALS CO., LTD.) (SFDA approval number: Z20026866) | Salvia miltiorrhiza Bunge [Lamiaceae];  Carthamus tinctorius L. [Asteraceae] | Y - Prepared according to People's Republic of China Pharmacopoeia | Y-HPLC |
| Wang 2016 | Danhong injection | Shandong Danhong Pharmaceutical Co., Ltd. (SHANDONG BUCHANG PHARMACEUTICALS CO., LTD.) (SFDA approval number: Z20026866) | Salvia miltiorrhiza Bunge [Lamiaceae];  Carthamus tinctorius L. [Asteraceae] | Y - Prepared according to People's Republic of China Pharmacopoeia | Y-HPLC |
| Xin 2015 | Danhong injection | Shandong Danhong Pharmaceutical Co., Ltd. (SHANDONG BUCHANG PHARMACEUTICALS CO., LTD.) (SFDA approval number: Z20026866) | Salvia miltiorrhiza Bunge [Lamiaceae];  Carthamus tinctorius L. [Asteraceae] | Y - Prepared according to People's Republic of China Pharmacopoeia | Y-HPLC |
| Xin and Ren, 2013 | Danhong injection | Shandong Danhong Pharmaceutical Co., Ltd. (SHANDONG BUCHANG PHARMACEUTICALS CO., LTD.) (SFDA approval number: Z20026866) | Salvia miltiorrhiza Bunge [Lamiaceae];  Carthamus tinctorius L. [Asteraceae] | Y - Prepared according to People's Republic of China Pharmacopoeia | Y-HPLC |
| Chen et al., 2008 | Breviscapine injection | Hengshan Hencer Pharmaceutical Co., Ltd. (SFDA approval number: NR) | Erigeron breviscapus (Vaniot) Hand.-Mazz. [Asteraceae] | Y - Prepared according to People's Republic of China Pharmacopoeia | Y-HPLC |
| He and Geng 2010 | Breviscapine injection | Shineway Pharmaceutical Group Ltd. (SFDA approval number: Z13020778) | Erigeron breviscapus (Vaniot) Hand.-Mazz. [Asteraceae] | Y - Prepared according to People's Republic of China Pharmacopoeia | Y-HPLC |
| Liu 2012b | Breviscapine injection | Support by Research Institution | Erigeron breviscapus (Vaniot) Hand.-Mazz. [Asteraceae] | Y - Prepared according to People's Republic of China Pharmacopoeia | Y-HPLC |
| Ma 2018 | Breviscapine injection | Heilongjiang Wusuli Jiang Pharmaceutical Co., Ltd. (SFDA approval number: Z23022213) | Erigeron breviscapus (Vaniot) Hand.-Mazz. [Asteraceae] | Y - Prepared according to People's Republic of China Pharmacopoeia | Y-HPLC |
| Qiao 2015 | Breviscapine injection | Support by Research Institution | Erigeron breviscapus (Vaniot) Hand.-Mazz. [Asteraceae] | Y - Prepared according to People's Republic of China Pharmacopoeia | Y-HPLC |
| Ren and Wu, 2006 | Breviscapine injection | Support by Research Institution | Erigeron breviscapus (Vaniot) Hand.-Mazz. [Asteraceae] | Y - Prepared according to People's Republic of China Pharmacopoeia | Y-HPLC |
| Wang and Lan, 2012 | Breviscapine injection | Kunming Longjin Pharmaceutical Co., Ltd. (SFDA approval number: Z20053907) | Erigeron breviscapus (Vaniot) Hand.-Mazz. [Asteraceae] | Y - Prepared according to People's Republic of China Pharmacopoeia | Y-HPLC |
| Wei and Tan, 2005 | Breviscapine injection | Harbin Sanlian Pharmaceutical Co., Ltd. (SFDA approval number: 030501) | Erigeron breviscapus (Vaniot) Hand.-Mazz. [Asteraceae] | Y - Prepared according to People's Republic of China Pharmacopoeia | Y-HPLC |
| Ye 2009 | Breviscapine injection | Support by Research Institution | Erigeron breviscapus (Vaniot) Hand.-Mazz. [Asteraceae] | Y - Prepared according to People's Republic of China Pharmacopoeia | Y-HPLC |
| Zhang et al., 2004 | Breviscapine injection | Support by Research Institution | Erigeron breviscapus (Vaniot) Hand.-Mazz. [Asteraceae] | Y - Prepared according to People's Republic of China Pharmacopoeia | Y-HPLC |
| Zhao et al., 2016 | Breviscapine injection | Kunming Longjin Pharmaceutical Co., Ltd.(SFDA approval number: Z20053907 ) | Erigeron breviscapus (Vaniot) Hand.-Mazz. [Asteraceae] | Y - Prepared according to People's Republic of China Pharmacopoeia | Y-HPLC |
| Zheng et al., 2006 | Breviscapine injection | Yunnan Biopharmaceutical Co., Ltd. (SFDA approval number: NR) | Erigeron breviscapus (Vaniot) Hand.-Mazz. [Asteraceae] | Y - Prepared according to People's Republic of China Pharmacopoeia | Y-HPLC |
| Chen et al., 2015 | Astragalus injection | Support by Research Institution | Astragalus mongholicus Bunge [Fabaceae] | Y - Prepared according to People's Republic of China Pharmacopoeia | Y-HPLC |
| Han et al., 2011 | Astragalus injection | Chengdu Diao Jiuhong Pharmaceutical Factory (SFDA approval number: Z51021775) | Astragalus mongholicus Bunge [Fabaceae] | Y - Prepared according to People's Republic of China Pharmacopoeia | Y-HPLC |
| Huang 2017 | Astragalus injection | HeiLongJiang ZBD Pharmaceutical Co., Ltd. (SFDA approval number: Z23020781) | Astragalus mongholicus Bunge [Fabaceae] | Y - Prepared according to People's Republic of China Pharmacopoeia | Y-HPLC |
| Huang et al., 2011 | Astragalus injection | Chengdu Diao Jiuhong Pharmaceutical Factory (SFDA approval number: Z51021775) | Astragalus mongholicus Bunge [Fabaceae] | Y - Prepared according to People's Republic of China Pharmacopoeia | Y-HPLC |
| Ji and Yin 2006 | Astragalus injection | Chengdu Diao Jiuhong Pharmaceutical Factory (SFDA approval number: Z51021775) | Astragalus mongholicus Bunge [Fabaceae] | Y - Prepared according to People's Republic of China Pharmacopoeia | Y-HPLC |
| Li 2006 | Astragalus injection | Support by Research Institution | Astragalus mongholicus Bunge [Fabaceae] | Y - Prepared according to People's Republic of China Pharmacopoeia | Y-HPLC |
| Luo 2014 | Astragalus injection | Chengdu Diao Jiuhong Pharmaceutical Factory (SFDA approval number: Z51021775) | Astragalus mongholicus Bunge [Fabaceae] | Y - Prepared according to People's Republic of China Pharmacopoeia | Y-HPLC |
| Tang et al., 2006 | Astragalus injection | Chengdu Diao Jiuhong Pharmaceutical Factory (SFDA approval number: Z51021775) | Astragalus mongholicus Bunge [Fabaceae] | Y - Prepared according to People's Republic of China Pharmacopoeia | Y-HPLC |
| Wu and Zhao, 2007 | Astragalus injection | Support by Research Institution | Astragalus mongholicus Bunge [Fabaceae] | Y - Prepared according to People's Republic of China Pharmacopoeia | Y-HPLC |
| Xu et al., 2008 | Astragalus injection | Chengdu Diao Jiuhong Pharmaceutical Factory (SFDA approval number: Z51021775) | Astragalus mongholicus Bunge [Fabaceae] | Y - Prepared according to People's Republic of China Pharmacopoeia | Y-HPLC |
| Xu 2015 | Astragalus injection | HeiLongJiang ZBD Pharmaceutical Co., Ltd. (SFDA approval number: Z23020781) | Astragalus mongholicus Bunge [Fabaceae] | Y - Prepared according to People's Republic of China Pharmacopoeia | Y-HPLC |
| Yang 2015 | Astragalus injection | Chengdu Diao Jiuhong Pharmaceutical Factory (SFDA approval number: Z51021775) | Astragalus mongholicus Bunge [Fabaceae] | Y - Prepared according to People's Republic of China Pharmacopoeia | Y-HPLC |
| Zhao 2017 | Astragalus injection | Shineway Pharmaceutical Group Ltd. (SFDA approval number: Z13020999) | Astragalus mongholicus Bunge [Fabaceae] | Y - Prepared according to People's Republic of China Pharmacopoeia | Y-HPLC |
| Zhao and Jing, 2015 | Astragalus injection | Chengdu Diao Jiuhong Pharmaceutical Factory (SFDA approval number: Z51021775) | Astragalus mongholicus Bunge [Fabaceae] | Y - Prepared according to People's Republic of China Pharmacopoeia | Y-HPLC |
| Zhao et al., 2015 | Astragalus injection | Chiatai Qingchunbao Pharmaceutical Co., Ltd.(SFDA approval number: Z33020178 ) | Astragalus mongholicus Bunge [Fabaceae] | Y - Prepared according to People's Republic of China Pharmacopoeia | Y-HPLC |
| Yao et al., 2002 | Astragalus injection | Chengdu Diao Jiuhong Pharmaceutical Factory (SFDA approval number: Z51021775) | Astragalus mongholicus Bunge [Fabaceae] | Y - Prepared according to People's Republic of China Pharmacopoeia | Y-HPLC |
| Bi et al., 2016 | Sodium tanshinone IIA sulfonate injection | Shanghai No. 1 Biochemical & Pharmaceutical Co., Ltd. (SFDA approval number: H31022558) OR Alberta Pharmaceutical Holdings (Tonghua) Co., Ltd. (SFDA approval number: H22026452) | Salvia miltiorrhiza Bunge [Lamiaceae] | Y - Prepared according to People's Republic of China Pharmacopoeia | Y-HPLC |
| Cao et al., 2014 | Sodium tanshinone IIA sulfonate injection | Shanghai No. 1 Biochemical & Pharmaceutical Co., Ltd. (SFDA approval number: H31022558) OR Alberta Pharmaceutical Holdings (Tonghua) Co., Ltd. (SFDA approval number: H22026452) | Salvia miltiorrhiza Bunge [Lamiaceae] | Y - Prepared according to People's Republic of China Pharmacopoeia | Y-HPLC |
| Hu 2017 | Sodium tanshinone IIA sulfonate injection | Alberta Pharmaceutical Holdings (Tonghua) Co., Ltd. (SFDA approval number: H22026452) | Salvia miltiorrhiza Bunge [Lamiaceae] | Y - Prepared according to People's Republic of China Pharmacopoeia | Y-HPLC |
| Li 2019 | Sodium tanshinone IIA sulfonate injection | Shanghai No. 1 Biochemical & Pharmaceutical Co., Ltd. (SFDA approval number: H31022558) | Salvia miltiorrhiza Bunge [Lamiaceae] | Y - Prepared according to People's Republic of China Pharmacopoeia | Y-HPLC |
| Li and Guo, 2017 | Sodium tanshinone IIA sulfonate injection | Shanghai No. 1 Biochemical & Pharmaceutical Co., Ltd. (SFDA approval number: H31022558) | Salvia miltiorrhiza Bunge [Lamiaceae] | Y - Prepared according to People's Republic of China Pharmacopoeia | Y-HPLC |
| Li 2016 | Sodium tanshinone IIA sulfonate injection | Shanghai No. 1 Biochemical & Pharmaceutical Co., Ltd. (SFDA approval number: H31022558) OR Alberta Pharmaceutical Holdings (Tonghua) Co., Ltd. (SFDA approval number: H22026452) | Salvia miltiorrhiza Bunge [Lamiaceae] | Y - Prepared according to People's Republic of China Pharmacopoeia | Y-HPLC |
| Li 2014 | Sodium tanshinone IIA sulfonate injection | Shanghai No. 1 Biochemical & Pharmaceutical Co., Ltd. (SFDA approval number: H31022558) OR Alberta Pharmaceutical Holdings (Tonghua) Co., Ltd. (SFDA approval number: H22026452) | Salvia miltiorrhiza Bunge [Lamiaceae] | Y - Prepared according to People's Republic of China Pharmacopoeia | Y-HPLC |
| Liu 2015 | Sodium tanshinone IIA sulfonate injection | Shanghai No. 1 Biochemical & Pharmaceutical Co., Ltd. (SFDA approval number: H31022558) | Salvia miltiorrhiza Bunge [Lamiaceae] | Y - Prepared according to People's Republic of China Pharmacopoeia | Y-HPLC |
| Liu 2018b | Sodium tanshinone IIA sulfonate injection | Shanghai No. 1 Biochemical & Pharmaceutical Co., Ltd. (SFDA approval number: H31022558) OR Alberta Pharmaceutical Holdings (Tonghua) Co., Ltd. (SFDA approval number: H22026452) | Salvia miltiorrhiza Bunge [Lamiaceae] | Y - Prepared according to People's Republic of China Pharmacopoeia | Y-HPLC |
| Ma et al., 2017 | Sodium tanshinone IIA sulfonate injection | Shanghai No. 1 Biochemical & Pharmaceutical Co., Ltd. (SFDA approval number: H31022558) | Salvia miltiorrhiza Bunge [Lamiaceae] | Y - Prepared according to People's Republic of China Pharmacopoeia | Y-HPLC |
| Qiu 2015 | Sodium tanshinone IIA sulfonate injection | Shanghai No. 1 Biochemical & Pharmaceutical Co., Ltd. (SFDA approval number: H31022558) | Salvia miltiorrhiza Bunge [Lamiaceae] | Y - Prepared according to People's Republic of China Pharmacopoeia | Y-HPLC |
| Shi et al., 2020 | Sodium tanshinone IIA sulfonate injection | Shanghai No. 1 Biochemical & Pharmaceutical Co., Ltd. (SFDA approval number: H31022558) | Salvia miltiorrhiza Bunge [Lamiaceae] | Y - Prepared according to People's Republic of China Pharmacopoeia | Y-HPLC |
| Tu 2017 | Sodium tanshinone IIA sulfonate injection | Shanghai No. 1 Biochemical & Pharmaceutical Co., Ltd. (SFDA approval number: H31022558) OR Alberta Pharmaceutical Holdings (Tonghua) Co., Ltd. (SFDA approval number: H22026452) | Salvia miltiorrhiza Bunge [Lamiaceae] | Y - Prepared according to People's Republic of China Pharmacopoeia | Y-HPLC |
| Wang et al., 2016 | Sodium tanshinone IIA sulfonate injection | Shanghai No. 1 Biochemical & Pharmaceutical Co., Ltd. (SFDA approval number: H31022558) | Salvia miltiorrhiza Bunge [Lamiaceae] | Y - Prepared according to People's Republic of China Pharmacopoeia | Y-HPLC |
| Wu 2013 | Sodium tanshinone IIA sulfonate injection | Shanghai No. 1 Biochemical & Pharmaceutical Co., Ltd. (SFDA approval number: H31022558) | Salvia miltiorrhiza Bunge [Lamiaceae] | Y - Prepared according to People's Republic of China Pharmacopoeia | Y-HPLC |
| Yu 2014 | Sodium tanshinone IIA sulfonate injection | Shanghai No. 1 Biochemical & Pharmaceutical Co., Ltd. (SFDA approval number: H31022558) OR Alberta Pharmaceutical Holdings (Tonghua) Co., Ltd. (SFDA approval number: H22026452) | Salvia miltiorrhiza Bunge [Lamiaceae] | Y - Prepared according to People's Republic of China Pharmacopoeia | Y-HPLC |
| Zhu et al., 2011 | Sodium tanshinone IIA sulfonate injection | Shanghai No. 1 Biochemical & Pharmaceutical Co., Ltd. (SFDA approval number: H31022558) | Salvia miltiorrhiza Bunge [Lamiaceae] | Y - Prepared according to People's Republic of China Pharmacopoeia | Y-HPLC |
| Zhu 2015 | Sodium tanshinone IIA sulfonate injection | Shanghai No. 1 Biochemical & Pharmaceutical Co., Ltd. (SFDA approval number: H31022558) OR Alberta Pharmaceutical Holdings (Tonghua) Co., Ltd. (SFDA approval number: H22026452) | Salvia miltiorrhiza Bunge [Lamiaceae] | Y - Prepared according to People's Republic of China Pharmacopoeia | Y-HPLC |
| Zou et al., 2013 | Sodium tanshinone IIA sulfonate injection | Shanghai No. 1 Biochemical & Pharmaceutical Co., Ltd. (SFDA approval number: H31022558) | Salvia miltiorrhiza Bunge [Lamiaceae] | Y - Prepared according to People's Republic of China Pharmacopoeia | Y-HPLC |
| Cai and Yan, 2015 | Ginkgo Leaf Extract and Dipyridamole injection | Support by Research Institution | Ginkgo biloba L. [Ginkgoaceae] | Y - Prepared according to People's Republic of China Pharmacopoeia | Y-HPLC |
| Chen and Feng, 2008 | Ginkgo Leaf Extract and Dipyridamole injection | Guizhou Yibai Pharmaceutical Co.,Ltd. (SFDA approval number: H52020032) | Ginkgo biloba L. [Ginkgoaceae] | Y - Prepared according to People's Republic of China Pharmacopoeia | Y-HPLC |
| He et al., 2006 | Ginkgo Leaf Extract and Dipyridamole injection | Guizhou Yibai Pharmaceutical Co.,Ltd. (SFDA approval number: H52020032) | Ginkgo biloba L. [Ginkgoaceae] | Y - Prepared according to People's Republic of China Pharmacopoeia | Y-HPLC |
| Li 2010 | Ginkgo Leaf Extract and Dipyridamole injection | Shanxi PUDE Pharmaceutical Co., Ltd. (SFDA approval number: H14023515)) | Ginkgo biloba L. [Ginkgoaceae] | Y - Prepared according to People's Republic of China Pharmacopoeia | Y-HPLC |
| Wang et al., 2014 | Ginkgo Leaf Extract and Dipyridamole injection | Guizhou Yibai Pharmaceutical Co.,Ltd. (SFDA approval number: H52020032) | Ginkgo biloba L. [Ginkgoaceae] | Y - Prepared according to People's Republic of China Pharmacopoeia | Y-HPLC |
| Ye and Zhang, 2011 | Ginkgo Leaf Extract and Dipyridamole injection | Shanxi PUDE Pharmaceutical Co., Ltd. (SFDA approval number: H14023515)) | Ginkgo biloba L. [Ginkgoaceae] | Y - Prepared according to People's Republic of China Pharmacopoeia | Y-HPLC |

*SFDA: State Food and Drug Administration

# S Table 4 Combined P-score of Biplots

| **Interventions** | **24-hour UPE combined with SBP** | | **24-hour UPE combined with DBP** | |
| --- | --- | --- | --- | --- |
|  | **Original network analysis results** | **Sensitivity analysis results** | **Original network analysis results** | **Sensitivity analysis results** |
| **ADs** | **0.00%** | **0.00%** | **0.00%** | **0.00%** |
| **AST+ADs** | **41.16%** | **46.80%** | **33.81%** | **32.76%** |
| **BRE+ADs** | **4.50%** | **7.04%** | **4.75%** | **8.64%** |
| **DH+ADs** | **6.50%** | **6.75%** | **8.50%** | **11.25%** |
| **GLED+ADs** | **88.00%** | **85.26%** | **72.00%** | **71.54%** |
| **SA+ADs** | **56.16%** | **40.02%** | **70.20%** | **52.20%** |
| **STS+ADs** | **38.34%** | **31.86%** | **44.73%** | **38.94%** |

SBP: Systolic blood pressure; DBP: Diastolic blood pressure.

ADs: antihypertensive drugs; SA: salvianolate; DH: danhong; BRE: breviscapine; AST: astragalus; STS: sodium tanshinone IIA sulfonate; GLED: ginkgo leaf extract and dipyridamole.

# S Table 5 League table of mALB and SCR

|  | **mALB (Mean Difference (95% credible interval)** | | | | | | |
| --- | --- | --- | --- | --- | --- | --- | --- |
| **SCR (Mean Difference (95% credible interval)** | ADs | -9.25  (-15.50, -2.99) | -40.09  (-51.50, -28.68) | -14.79  (-19.90, -9.68) |  | -23.87  (-33.64, -14.11) | -14.17  (-20.77, -7.56) |
|  | -17.70  (-30.68, -4.72) | AST+ADs | -30.85  (-43.86, -17.83) | -5.55  (-13.63, 2.53) |  | -14.63  (-26.23, -3.03) | -4.92  (-14.02, 4.18) |
|  | -26.48  (-39.18, -13.78) | -8.78  (-26.94, 9.38) | BRE+ADs | 25.30  (12.80, 37.80) |  | 16.22  (1.20, 31.24) | 25.92  (12.74, 39.10) |
|  | -26.07  (-41.30, -10.84) | -8.37  (-28.38, 11.64) | 0.41  (-19.42, 20.24) | DH+ADs |  | -9.08  (-20.10, 1.94) | 0.63  ( -7.72, 8.98) |
|  | -21.51  (-39.53, -3.50) | -3.81  (-26.01, 18.39) | 4.97  (-17.07, 27.01) | 4.56  (-19.03, 28.15) | GLED+ADs |  |  |
|  | -17.13  (-33.61, -0.65) | 0.57  (-20.41, 21.55) | 9.35  (-11.46, 30.16) | 8.94  (-13.50, 31.38) | 4.38  (-20.03, 28.80) | SA+ADs | 9.70  ( -2.08, 21.49) |
|  | -20.42  (-29.49, -11.35) | -2.72  (-18.55, 13.12) | 6.06  ( -9.54, 21.67) | 5.66  (-12.07, 23.38) | 1.09  (-19.07, 21.26) | -3.29  (-22.10, 15.53) | STS+ADs |

ADs: antihypertensive drugs; SA: salvianolate; DH: danhong; BRE: breviscapine; AST: astragalus; STS: sodium tanshinone IIA sulfonate; GLED: ginkgo leaf extract and dipyridamole.

mALB: micro-albuminuria; SCR: serum creatinine.

Black number with green background represented significance difference, whereas white number with gray background represented no significance difference.

# S Table 6 League table of BUN and CCR

|  | **BUN (Mean Difference (95% credible interval)** | | | | | | |
| --- | --- | --- | --- | --- | --- | --- | --- |
| **CCR (Mean Difference (95% credible interval)** | ADs | -1.22  (-2.29, -0.15) | -1.89  (-2.93, -0.85) | -1.42  (-2.79, -0.05) | -1.36  (-2.54, -0.19) | -1.93  (-3.78, -0.08) | -1.95  (-3.40, -0.49) |
|  | 9.38  (-20.72,39.48) | AST+ADs | -0.67  (-2.16, 0.82) | -0.20  (-1.94, 1.53) | -0.14  (-1.73, 1.44) | -0.71  (-2.84, 1.42) | -0.72  (-2.53, 1.08) |
|  | 7.21  (-10.10,24.52) | -2.17  (-36.89,32.55) | BRE+ADs | 0.47  (-1.25, 2.18) | 0.52  (-1.04, 2.09) | -0.04  (-2.16, 2.08) | -0.06  (-1.84, 1.73) |
|  | 18.78  (5.42,32.13) | 9.40  (-23.53,42.32) | 11.56  (-10.30,33.43) | DH+ADs | 0.06  (-1.75, 1.86) | -0.51  (-2.81, 1.79) | -0.52  (-2.52, 1.48) |
|  | 12.33  ( -5.34,29.99) | 2.95  (-31.95,37.85) | 5.12  (-19.62,29.85) | -6.45  (-28.59,15.70) | GLED+ADs | -0.57  (-2.75, 1.62) | -0.58  (-2.45, 1.29) |
|  |  |  |  |  |  | SA+ADs | -0.01  (-2.36, 2.33) |
|  |  |  |  |  |  |  | STS+ADs |

ADs: antihypertensive drugs; SA: salvianolate; DH: danhong; BRE: breviscapine; AST: astragalus; STS: sodium tanshinone IIA sulfonate; GLED: ginkgo leaf extract and dipyridamole.

BUN: blood urea nitrogen; CCR: creatinine clearance rate.

Black number with green background represented significance difference, whereas white number with gray background represented no significance difference.

# S Table 7 League table of β2-MG and Adverse events

|  | **β2-MG (Mean Difference (95% credible interval)** | | | | | |
| --- | --- | --- | --- | --- | --- | --- |
| **AE**  **(Odds ratios (95% credible interval)** | ADs | -0.23  (-0.37, -0.09) | -0.12  (-0.28, 0.05) | -0.10  (-0.20, 0.00) | -0.15  (-0.31, 0.00) |  |
|  | 0.79  (0.19, 3.22) | AST+ADs | 0.11  (-0.11, 0.33) | 0.12  (-0.05, 0.30) | 0.07  (-0.14, 0.28) |  |
|  | 1.54  (0.70, 3.42) | 1.96  (0.39, 9.93) | BRE+ADs | 0.01  (-0.18, 0.21) | -0.04  (-0.26, 0.19) |  |
|  | 2.62  (0.49,13.89) | 3.33  (0.37,29.61) | 1.69 (0.27,10.76) | DH+ADs | -0.05  (-0.23, 0.14) |  |
|  |  |  |  |  | STS+ADs |  |
|  | 4.06 (0.44,37.47) | 5.17 (0.37,71.86) | 2.63 (0.25,27.86) | 1.55 (0.10,25.02) |  | SA+ADs |

ADs: antihypertensive drugs; SA: salvianolate; DH: danhong; BRE: breviscapine; AST: astragalus; STS: sodium tanshinone IIA sulfonate.

β2-MG: β2-microglobulin; AE: adverse events.

Black number with green background represented significance difference, whereas white number with gray background represented no significance difference.

# S Table 8 Adverse events

| **SA** | | | | | |
| --- | --- | --- | --- | --- | --- |
| **Author Year** | **Interventions** | **Total adverse events** | **Cereal third transaminase high** | **Dizziness** | **Conscious fever** |
| Tian 2019 | SA+ADs | 1 | 1 |  |  |
| Wang 2015a | SA+ADs | 2 |  | 1 | 1 |

| **DH** | | | | |
| --- | --- | --- | --- | --- |
| **Author Year** | **Interventions** | **Total adverse events** | **Dizziness** | **Cough** |
| Li 2012 | DH+ADs | 2 |  | 2 |
| Meng 2014 | DH+ADs | 1 | 1 |  |
| Peng 2013 | DH+ADs | 1 | 1 |  |

| **BRE** | | | | | | |
| --- | --- | --- | --- | --- | --- | --- |
| **Author Year** | **Interventions** | **Total adverse events** | **Dizziness** | **Cough** | **Facial flushing** | **Facial flushing** |
| Chen 2008a | BRE+ADs | 1 |  | 1 |  |  |
| He 2010 | BRE+ADs | 5 |  | 5 |  |  |
| Ren 2006 | BRE+ADs | 2 |  | 2 |  |  |
| Wang 2012 | BRE+ADs | 1 |  | 1 |  |  |
| Wei 2005 | BRE+ADs | 1 |  |  | 1 | 1 |
| Ye 2009 | BRE+ADs | 1 | 1 |  |  |  |
| Zhang 2004 | BRE+ADs | 3 |  | 3 |  |  |
| Zheng 2006 | BRE+ADs | 2 |  | 2 |  |  |

| **AST** | | | |
| --- | --- | --- | --- |
| **Author Year** | **Interventions** | **Total adverse events** | **Headache** |
| Huang 2017 | AST+ADs | 2 (No detailed information) |  |
| Luo 2014 | AST+ADs | 2 | 2 |

| **ADs** | | | | | |
| --- | --- | --- | --- | --- | --- |
| **Author Year** | **Interventions** | **Total adverse events** | **Headache** | **Distention of head** | **Cough** |
| Li 2012 | ADs | 1 |  |  | 1 |
| Chen 2008a | ADs | 1 |  |  | 1 |
| He 2010 | ADs | 3 |  |  | 3 |
| Ren 2006 | ADs | 1 |  | 1 |  |
| Wang 2012 | ADs | 2 |  |  | 2 |
| Zhang 2004 | ADs | 2 |  |  | 2 |
| Zheng 2006 | ADs | 1 |  | 1 |  |
| Huang 2017 | ADs | 4 |  |  |  |
| Luo 2014 | ADs | 1 | 1 |  |  |

ADs: antihypertensive drugs; SA: salvianolate; DH: danhong; BRE: breviscapine; AST: astragalus injection; STS: sodium tanshinone IIA sulfonate; GLED: ginkgo leaf extract and dipyridamole.

# S Table 9 The Meta-regression for Primary Outcomes

| **24-hour UPE** | | | | | | | | |
| --- | --- | --- | --- | --- | --- | --- | --- | --- |
|  | **Interventions** | **Mean** | **SD** | **2.50%** | **25%** | **50%** | **75%** | **97.50%** |
| **Usage of ADs** | AST + ADs | *0.51* | *0.1* | 0.21 | 0.46 | 0.53 | 0.58 | 0.68 |
|  | BRE + ADs | *0.39* | *1.51* | -1.53 | -1 | 0.28 | 1.45 | 3.43 |
|  | DH + ADs | *0.08* | *1.35* | -2.97 | -0.51 | 0.39 | 0.88 | 2.22 |
|  | GLED + ADs | *-1.12* | *1.42* | -3.50 | -2.21 | -1.41 | -0.01 | 1.58 |
|  | SA + ADs | *0.30* | *2.29* | -5.44 | -0.2 | 0.91 | 2.14 | 2.85 |
|  | STS + ADs | *0.21* | *1.1* | -1.65 | -0.67 | 0.22 | 1.07 | 2.29 |
| **Types of ADs** | AST + ADs | *-0.12* | *0.03* | -0.20 | -0.13 | -0.11 | -0.09 | -0.06 |
|  | BRE + ADs | *0.50* | *0.8* | -0.73 | -0.12 | 0.32 | 1.03 | 2.13 |
|  | DH + ADs | *-1.00* | *2.17* | -4.43 | -2.57 | -1.45 | 0.53 | 3.53 |
|  | GLED + ADs | *-0.50* | *0.99* | -2.53 | -1.03 | -0.51 | 0.18 | 1.51 |
|  | SA + ADs | *-2.61* | *2.39* | -7.35 | -4.58 | -1.78 | -1.07 | 1.23 |
|  | STS + ADs | *-2.78* | *2.54* | -7.1 | -5.44 | -2.14 | -0.49 | 1.03 |
| **Sample size** | AST + ADs | *0.00* | *0.01* | -0.01 | 0 | 0 | 0 | 0.01 |
|  | BRE + ADs | *0.00* | *0.07* | -0.13 | -0.06 | 0 | 0.06 | 0.12 |
|  | DH + ADs | *0.45* | *0.33* | -0.22 | 0.2 | 0.52 | 0.69 | 0.99 |
|  | GLED + ADs | *0.01* | *0.03* | -0.06 | -0.01 | 0.02 | 0.03 | 0.05 |
|  | SA + ADs | *-1.43* | *1.1* | -2.82 | -2.18 | -1.74 | -1.12 | 1.08 |
|  | STS + ADs | *0.00* | *0* | -0.01 | 0 | 0 | 0 | 0.01 |
| **Male** | AST + ADs | *0.00* | *0.01* | -0.02 | 0 | 0 | 0.01 | 0.01 |
|  | BRE + ADs | *0.00* | *0.14* | -0.22 | -0.11 | 0.01 | 0.12 | 0.25 |
|  | DH + ADs | *-0.33* | *0.71* | -1.41 | -0.96 | -0.3 | -0.03 | 1.31 |
|  | GLED + ADs | *-0.03* | *0.18* | -0.29 | -0.16 | -0.05 | 0.07 | 0.3 |
|  | SA + ADs | *0.65* | *0.45* | -0.4 | 0.4 | 0.76 | 0.97 | 1.27 |
|  | STS + ADs | *0.00* | *0.01* | -0.01 | -0.01 | 0 | 0 | 0.02 |
| **Mean age** | AST + ADs | *0.01* | *0.01* | 0 | 0.01 | 0.02 | 0.02 | 0.02 |
|  | BRE + ADs | *0.01* | *0.04* | -0.04 | -0.03 | 0.01 | 0.04 | 0.07 |
|  | DH + ADs | *-0.04* | *0.55* | -0.72 | -0.48 | -0.09 | 0.2 | 1.42 |
|  | GLED + ADs | *0.01* | *0.06* | -0.06 | -0.04 | -0.02 | 0.04 | 0.13 |
|  | SA + ADs | *-2.16* | *3.28* | -9.4 | -3 | -2.18 | -0.49 | 3.57 |
|  | STS + ADs | *-0.01* | *0.01* | -0.02 | -0.01 | -0.01 | 0 | 0 |
| **Course of disease** | AST + ADs | *-0.02* | *0.02* | -0.04 | -0.03 | -0.02 | -0.01 | 0.02 |
|  | BRE + ADs | *-0.14* | *0.3* | -0.69 | -0.36 | -0.16 | 0.17 | 0.27 |
|  | DH + ADs | *0.89* | *1.89* | -2.49 | -1.04 | 1.31 | 2.25 | 3.75 |
|  | GLED + ADs | *-0.08* | *0.3* | -0.47 | -0.28 | -0.15 | 0.01 | 0.55 |
|  | SA + ADs | *3.88* | *8.93* | -4.12 | -2.88 | 1.47 | 5.37 | 24.7 |
|  | STS + ADs | *0.00* | *0.01* | -0.02 | -0.01 | 0 | 0.01 | 0.03 |
| **Course of treatment** | AST + ADs | *-0.04* | *0.03* | -0.10 | -0.06 | -0.04 | -0.02 | 0.01 |
|  | BRE + ADs | *-0.03* | *2.79* | -6.29 | -1 | 0.48 | 1.89 | 6 |
|  | DH + ADs | *2.45* | *4.82* | -1.12 | -0.84 | 0.26 | 2 | 14.95 |
|  | GLED + ADs | *-1.45* | *3.04* | -7.73 | -3.18 | -0.09 | 0.53 | 1.81 |
|  | SA + ADs | *1.34* | *2.2* | -1.51 | -0.98 | 1.98 | 3.38 | 4.7 |
|  | STS + ADs | *0.08* | *0.05* | -0.01 | 0.05 | 0.08 | 0.11 | 0.2 |
| **SBP** | | | | | | | | |
|  | **Interventions** | **Mean** | **SD** | **2.50%** | **25%** | **50%** | **75%** | **97.50%** |
| **Usage of ADs** | AST+ADs | *12.51* | *5.06* | 1.75 | 9.36 | 12.75 | 15.95 | 21.75 |
|  | BRE+ADs | *-0.93* | *8.2* | -16.73 | -6.48 | -0.98 | 4.47 | 15.29 |
|  | DH+ADs | *1.5* | *9.88* | -17.76 | -5.16 | 1.45 | 8.08 | 21.1 |
|  | GLED+ADs | *0.13* | *9.97* | -19.37 | -6.61 | 0.23 | 6.82 | 19.59 |
|  | SA+ADs | *0.2* | *9.91* | -19.36 | -6.41 | 0.2 | 6.81 | 19.55 |
|  | STS+ADs | *2.16* | *9.71* | -16.9 | -4.39 | 2.21 | 8.71 | 21.12 |
| **Types of ADs** | AST+ADs | *-3.25* | *2.47* | -7.87 | -4.9 | -3.34 | -1.68 | 1.93 |
|  | BRE+ADs | *-3.02* | *5.49* | -13.76 | -6.69 | -3 | 0.71 | 7.64 |
|  | DH+ADs | *-8.56* | *7.08* | -22.5 | -13.3 | -8.59 | -3.91 | 5.57 |
|  | GLED+ADs | *0.01* | *10.08* | -19.85 | -6.71 | 0.04 | 6.87 | 19.59 |
|  | SA+ADs | *0.73* | *9.98* | -18.71 | -6.03 | 0.74 | 7.46 | 20.18 |
|  | STS+ADs | *-7.43* | *4.47* | -15.84 | -10.4 | 7.61 | -4.63 | 1.94 |
| **Sample size** | AST+ADs | *0.59* | *0.24* | 0.1 | 0.43 | 0.59 | 0.75 | 1.05 |
|  | BRE+ADs | *0.92* | *0.41* | 0.08 | 0.65 | 0.92 | 1.18 | 1.71 |
|  | DH+ADs | *0.05* | *0.27* | -0.49 | -0.13 | 0.05 | 0.23 | 0.57 |
|  | GLED+ADs | *0.12* | *4.41* | -8.55 | -2.85 | 0.12 | 3.07 | 8.78 |
|  | SA+ADs | *0.64* | *3.5* | -6.26 | -1.72 | 0.67 | 3 | 7.36 |
|  | STS+ADs | *-0.02* | *0.13* | -0.29 | -0.1 | -0.02 | 0.06 | 0.24 |
| **Male** | AST+ADs | *-0.64* | *0.36* | -1.33 | -0.88 | -0.65 | -0.41 | 0.08 |
|  | BRE+ADs | *-1.33* | *0.7* | -2.71 | -1.79 | -1.34 | -0.88 | 0.08 |
|  | DH+ADs | *0.18* | *0.19* | -0.2 | 0.06 | 0.18 | 0.31 | 0.56 |
|  | GLED+ADs | *0.4* | *2.51* | -4.56 | -1.26 | 0.4 | 2.09 | 5.29 |
|  | SA+ADs | *-0.09* | *6.79* | -13.26 | -4.74 | -0.14 | 4.55 | 13.14 |
|  | STS+ADs | *0.02* | *0.25* | -0.49 | -0.14 | 0.02 | 0.18 | 0.53 |
| **Mean age** | AST+ADs | *0.57* | *0.25* | 0.06 | 0.41 | 0.57 | 0.73 | 1.06 |
|  | BRE+ADs | *-0.1* | *0.15* | -0.39 | -0.2 | -0.1 | -0.01 | 0.2 |
|  | DH+ADs | *0.43* | *0.76* | -1.06 | -0.08 | 0.43 | 0.95 | 1.91 |
|  | GLED+ADs | *0.14* | *8.51* | -16.69 | -5.58 | 0.14 | 5.75 | 16.84 |
|  | SA+ADs | *1.59* | *6.34* | -10.88 | -2.64 | 1.69 | 5.91 | 13.84 |
|  | STS+ADs | *-0.18* | *0.14* | -0.46 | -0.27 | -0.18 | -0.09 | 0.1 |
| **Course of disease** | AST+ADs | *0.01* | *0.86* | -0.24 | 0.81 | 1.33 | 1.89 | 3.2 |
|  | BRE+ADs | *0.02* | *0.89* | -0.6 | 0.55 | 1.14 | 1.75 | 2.88 |
|  | DH+ADs | *0.18* | *1.44* | -2.64 | -0.79 | 0.18 | 1.15 | 3.01 |
|  | GLED+ADs | *-0.06* | *10* | -19.71 | -6.71 | -0.14 | 6.7 | 19.55 |
|  | SA+ADs | *0.07* | *5.32* | -13.51 | -6.73 | -3.11 | 0.54 | 7.37 |
|  | STS+ADs | *0.53* | *0.35* | -1.29 | -0.75 | -0.52 | -0.3 | 0.12 |
| **Course of treatment** | AST+ADs | *0.34* | *1.45* | -3.29 | -1.28 | -0.32 | 0.62 | 2.44 |
|  | BRE+ADs | *1.33* | *3.26* | -7.78 | -3.43 | -1.35 | 0.76 | 5.2 |
|  | DH+ADs | *2.69* | *2.69* | -6.52 | -3.12 | -1.32 | 0.47 | 3.99 |
|  | GLED+ADs | *0.16* | *10.01* | -19.54 | -6.66 | 0.18 | 6.95 | 19.74 |
|  | SA+ADs | *1.31* | *9.51* | -17.37 | -5.07 | 1.31 | 7.69 | 20.14 |
|  | STS+ADs | *0.62* | *0.62* | -0.64 | 0.17 | 0.56 | 0.96 | 1.83 |
| **DBP** | | | | | | | | |
|  | **Interventions** | **Mean** | **SD** | **2.50%** | **25%** | **50%** | **75%** | **97.50%** |
| **Usage of ADs** | AST+ADs | *0.02* | *7.45* | -14.36 | -5.04 | -0.16 | 5.05 | 14.64 |
|  | BRE+ADs | *3.38* | *7.47* | -11.50 | -1.59 | 3.51 | 8.45 | 17.85 |
|  | DH+ADs | *0.06* | *9.74* | -19.14 | -6.51 | 0.05 | 6.68 | 19.13 |
|  | GLED+ADs | *0.05* | *10.02* | -19.36 | -6.67 | -0.06 | 6.79 | 19.86 |
|  | SA+ADs | *0.37* | *10.01* | -19.35 | -6.29 | 0.49 | 7.09 | 19.81 |
|  | STS+ADs | *0.82* | *9.77* | -17.88 | -5.89 | 0.71 | 7.27 | 20.34 |
| **Types of ADs** | AST+ADs | *1.01* | *4.16* | -7.10 | -1.82 | 1.08 | 3.81 | 9.04 |
|  | BRE+ADs | *0.11* | *6.14* | -11.80 | -4.00 | 0.08 | 4.14 | 12.46 |
|  | DH+ADs | *-0.42* | *7.41* | -14.93 | -5.36 | -0.48 | 4.51 | 14.45 |
|  | GLED+ADs | *-0.10* | *10.06* | -19.70 | -6.90 | -0.04 | 6.73 | 19.53 |
|  | SA+ADs | *1.53* | *9.52* | -16.99 | -4.92 | 1.57 | 8.02 | 20.15 |
|  | STS+ADs | *0.97* | *4.69* | -8.11 | -2.13 | 0.92 | 4.04 | 10.26 |
| **Sample size** | AST+ADs | *-0.52* | *0.67* | -1.81 | -0.97 | -0.53 | -0.08 | 0.78 |
|  | BRE+ADs | *0.48* | *0.35* | -0.22 | 0.25 | 0.49 | 0.71 | 1.14 |
|  | DH+ADs | *-0.01* | *0.28* | -0.58 | -0.20 | -0.01 | 0.18 | 0.54 |
|  | GLED+ADs | *0.18* | *4.99* | -9.79 | -3.14 | 0.20 | 3.56 | 10.05 |
|  | SA+ADs | *-0.59* | *3.23* | -6.82 | -2.81 | -0.62 | 1.62 | 5.76 |
|  | STS+ADs | *-0.12* | *0.11* | -0.33 | -0.19 | -0.12 | -0.05 | 0.09 |
| **Male** | AST+ADs | *1.34* | *1.47* | -1.55 | 0.36 | 1.35 | 2.33 | 4.20 |
|  | BRE+ADs | *-0.82* | *0.55* | -1.88 | -1.19 | -0.83 | -0.47 | 0.31 |
|  | DH+ADs | *0.00* | *0.19* | -0.38 | -0.12 | 0.00 | 0.13 | 0.39 |
|  | GLED+ADs | *-0.13* | *5.69* | -11.39 | -3.87 | -0.13 | 3.65 | 11.03 |
|  | SA+ADs | *1.37* | *7.35* | -13.17 | -3.62 | 1.47 | 6.37 | 15.65 |
|  | STS+ADs | *0.21* | *0.20* | -0.20 | 0.08 | 0.21 | 0.33 | 0.59 |
| **Mean age** | AST+ADs | *1.12* | *1.05* | -0.94 | 0.43 | 1.13 | 1.83 | 3.18 |
|  | BRE+ADs | *0.34* | *0.41* | -0.46 | 0.06 | 0.33 | 0.61 | 1.16 |
|  | DH+ADs | *-0.11* | *0.71* | -1.53 | -0.59 | -0.11 | 0.37 | 1.29 |
|  | GLED+ADs | *-0.04* | *8.98* | -17.66 | -6.20 | -0.05 | 6.06 | 17.61 |
|  | SA+ADs | *-1.37* | *4.66* | -10.42 | -4.56 | -1.41 | 1.77 | 7.88 |
|  | STS+ADs | *0.10* | *0.13* | -0.16 | 0.02 | 0.10 | 0.19 | 0.37 |
| **Course of disease** | AST+ADs | *2.36* | *2.55* | -2.63 | 0.65 | 2.43 | 4.08 | 7.31 |
|  | BRE+ADs | *1.54* | *1.23* | -0.85 | 0.71 | 1.53 | 2.36 | 3.99 |
|  | DH+ADs | *0.09* | *0.98* | -1.89 | -0.56 | 0.10 | 0.74 | 2.00 |
|  | GLED+ADs | *-0.18* | *7.50* | -15.02 | -5.18 | -0.08 | 4.78 | 14.58 |
|  | SA+ADs | *0.43* | *9.76* | -18.82 | -6.15 | 0.41 | 7.08 | 19.36 |
|  | STS+ADs | *-0.37* | *0.34* | -1.05 | -0.59 | -0.37 | -0.15 | 0.32 |
| **Course of treatment** | AST+ADs | *-4.32* | *2.77* | -9.76 | -6.19 | -4.37 | -2.48 | 1.10 |
|  | BRE+ADs | *1.01* | *3.05* | -5.03 | -1.02 | 1.00 | 3.05 | 6.87 |
|  | DH+ADs | *0.18* | *2.50* | -4.73 | -1.49 | 0.19 | 1.83 | 5.09 |
|  | GLED+ADs | *-0.22* | *9.98* | -19.59 | -6.92 | -0.30 | 6.64 | 19.28 |
|  | SA+ADs | *3.17* | *8.08* | -12.72 | -2.25 | 3.19 | 8.50 | 19.31 |
|  | STS+ADs | *0.43* | *0.55* | -0.67 | 0.08 | 0.43 | 0.77 | 1.52 |

# S File 1 Protocol amendments

We adjusted the statistical methodology during the research process, which frequentist network meta-analysis were conducted in order to have a robust result. The Bayesian and frequentist results are generally consistent, and both of them are used widely in network meta-analysis. Technically, it doesn’t matter which method is used. However, the computation speed of frequentist method is much faster and more convenient than the Bayesian one. We finally chose the frequentist one to calculate.

# S File 2 The Detailed Search Strategy

**The Pubmed database Search Strategy**

#1: ((((((((hypertensive nephropathy[MeSH Terms]) OR (hypertensive nephropathy[Title/Abstract])) OR (high blood pressure with chronic kidney diseases[Title/Abstract])) OR (hypertension with chronic kidney diseases[Title/Abstract])) OR (high blood pressure with CKD[Title/Abstract])) OR (hypertension with CKD[Title/Abstract])) OR (Hypertension with renal injury[Title/Abstract])) OR (hypertensive renal damage[Title/Abstract])) OR (hypertensive proteinuria[Title/Abstract])

Results 63843

#2: (((((((traditional Chinese medicine injection[MeSH Terms]) OR (traditional Chinese medicine injection[Title/Abstract])) OR (Salvianolate[Title/Abstract])) OR (Danhong[Title/Abstract])) OR (Breviscapine[Title/Abstract])) OR (Astragalus [Title/Abstract])) OR (Sodium tanshinone IIA sulfonate[Title/Abstract])) OR (Ginkgo Leaf Extract and Dipyridamole[Title/Abstract])

Results 4428

#3: (((((((((randomized controlled study[Title/Abstract]) OR randomized controlled trial[Title/Abstract]) OR randomized trial[Title/Abstract]) OR randomized study[Title/Abstract]) OR randomized placebo-controlled study[Title/Abstract]) OR randomized parallel-group study[Title/Abstract]) OR controlled clinical trial[Title/Abstract]) OR multicenter study[Title/Abstract]) OR double-blinded controlled study[Title/Abstract])

Results 202543

#4：#1 AND #2 AND #3

Results **0**

**The Cochrane library database Search Strategy**

#1: (high blood pressure with chronic kidney diseases OR hypertension with chronic kidney diseases OR high blood pressure with CKD OR hypertension with CKD OR Hypertension with renal injury OR hypertensive renal damage OR hypertensive nephropathy OR hypertensive proteinuria):ti,ab,kw

Results 2514

#2: (traditional Chinese medicine injection OR Salvianolate OR Danhong OR Breviscapine OR Astragalus OR Sodium tanshinone IIA sulfonate OR Ginkgo Leaf Extract and Dipyridamole):ti,ab,kw

Results 906

#3: (randomized controlled study or randomized controlled trial or randomized trial or randomized study or randomized placebo-controlled study or randomized parallel-group study or controlled clinical trial or multicenter study or double-blinded controlled study):ti,ab,kw

Results 894596

#:4：#1 AND #2 AND#3

Results 3

**The Embase database Search Strategy**

#1: 'high blood pressure with chronic kidney diseases':ab,ti OR 'hypertension with chronic kidney diseases':ab,ti OR 'high blood pressure with ckd':ab,ti OR 'hypertension with ckd':ab,ti OR 'hypertension with renal injury':ab,ti OR 'hypertensive renal damage':ab,ti OR 'hypertensive nephropathy':ab,ti OR 'hypertensive proteinuria':ab,ti

Results 913

#2: 'traditional chinese medicine injection':ab,ti OR salvianolate:ab,ti OR danhong:ab,ti OR breviscapine:ab,ti OR Astragalus:ab,ti OR 'sodium tanshinone iia sulfonate':ab,ti OR ('ginkgo leaf extract and dipyridamole':ab,ti)

Results 4709

#3: 'randomized controlled study':ab,ti OR 'randomized controlled trial':ab,ti OR 'randomized trial':ab,ti OR 'randomized study':ab,ti OR 'randomized placebo-controlled study':ab,ti OR 'randomized parallel-group study':ab,ti OR 'controlled clinical trial':ab,ti OR 'multicenter study':ab,ti OR 'double-blinded controlled study':ab,ti

Results 28112

#4: #1 AND #2 AND #3

Results 0

**Web of Science**

#1: (high blood pressure with chronic kidney diseases OR hypertension with chronic kidney diseases OR high blood pressure with CKD OR hypertension with CKD OR Hypertension with renal injury OR hypertensive renal damage OR hypertensive nephropathy OR hypertensive proteinuria):ti,ab,kw

Results 23865

#2: (traditional Chinese medicine injection OR Salvianolate OR Danhong OR Breviscapine OR Astragalus OR Sodium tanshinone IIA sulfonate OR Ginkgo Leaf Extract and Dipyridamole):ti,ab,kw

Results 7038

#3: (randomized controlled study or randomized controlled trial or randomized trial or randomized study or randomized placebo-controlled study or randomized parallel-group study or controlled clinical trial or multicenter study or double-blinded controlled study):ti,ab,kw

Results 910546

#4: #1 AND #2 AND #3

Results 4

**Results of the references of included studies and reviews and Register the website of clinical trials**

References of included studied Results =0

References of reviews Results =0

Chinese Clinical Trial Registry (http://www.chictr.org.cn ) Results =0

Clinicaltrials.gov Results =0

# S File 3 Reference of Included Studies

Bi, X. J.,Wang, L. N.,Xin, H. J.,Wang, W.,Zhang, Y. R.,Qin, N., et al. (2016). Sodium tanshinon Ⅱ A silate sodium combined with irbesartan in the treatment of hypertension with renal damage and its effect on serum levels of PCX and PICP. Northwest Pharmaceutical Journal. 31, 403-405

Cao, G. Q.,Xu, X. H. and Han, R. Q. (2014). Efficacy analysis of irbesartan combined with tanshinone ⅡA sulfonate in the treatment of hypertension complicated with renal damage. Chinese Journal of Clinical Rational Drug Use. 7, 46-47

Chen, C. P. and Feng, Q. B. (2008). Clinical observation of 35 cases of hypertensive nephropathy treated with Xingding Injection and valsartan. Guiding Journal of Traditional Chinese Medicine and Pharmacy. 14, 47+57

Chen, X. J.,Wang, H. W. and Chen, L. (2015). Influence of huangqi injection on antioxidant capacity and vascular elasticity of patients with renal hypertension. Journal of Hainan Medical University. 21, 375-377

Chen, Y. J.,Lin, L. H.,Huang, X. T. and Xiao, H. (2008). Observation of therapeutic effect of breviscapine combined with benazepril on hypertensive nephropathy. International Medicine and Health Guidance News. 14, 69-71

Cai, J. Y. and Yan, X. Y. (2015). Clinical study of Yin Xing Da Mo injection combined with benazepril in the treatment of elderly patients hypertension renal damage. Chinese Community Doctors. 31, 25-26

Ding, J. S. (2017). Effect of valsartan and salvianolate on pressure and renal function in the treatment of hypertensive nephropathy. Clinical Research and Practice. 2, 22-23

Han, W. L.,Wang, S. H. and Li, Y. (2011). Effect of irbesartan combined with astragalus injection on renal damage of essential hypertension. Hebei Journal of Traditional Chinese Medicine. 33, 1505-1506

He, B. L. and Geng, L. J. (2010). Effect of breviscapine combined with captopril on early renal damage of hypertension. Chinese Journal of Misdiagnostics. 10, 2329-2330

He, D. M.,Zhuo, S. H.,Chen, Z. J.,Chen, M.,Wu, C. F.,Xie, H. B., et al. (2006). Effect of Xingding Injection on Hypertensive Kidney Injury with Mild to Moderate Proteinuria. Chinese Journal of Integrated Traditional and Western Nephrology. 07, 415-417

Hu, S. Y. (2017). Effects of tanshinone combined with valsartan on renal function and inflammatory response in patients with hypertensive nephropathy. Research of Integrated Traditional Chinese and Western Medicine. 9, 239-240

Huang, L. Y. (2017). Effect and mechanism of astragalus membranaceus combined with irberartan in the treatment of hypertensive nephropathy. Journal of Modern Medicine & Health. 33, 3456-3458

Huang, X. Z.,Zhang, D. W.,Fan, Y. P.,Chen, X. L. and Shi, H. (2011). Effect of astragalus injection combined with irbesartan on early renal damage of hypertension. Shaanxi Medical Journal. 40, 1663-1664

Ji, Y. and Yin, W. H. (2006). Effect of Astragalus Injection on Kidney Injury in Hypertension. Journal of Emergency in Traditional Chinese Medicine. 15, 1237-1238

Li, C. X. (2012). Observation on effect of perindopril plus danhong injection on early renal damage of hypertension. People's Military Surgeon. 55, 424-425

Li, G. L.,Liu, Z. Y.,Cui, W. N. and Wang, Y. R. (2010). Clinical Observation on Danhong Injection with Benazepril f or Elderly Hypertensive Patients with Early Kidney Demage. Chinese Journal of Integrative Medicine on Cardio-Cerebrovascular Disease. 8, 7-8

Li, J. (2019). Efficacy of Losartan Combined with Tanshinone Injection in the Treatment of Hypertensive Renal Damage. Journal of Medical Information. 32, 138-140

Li, J. L. (2010). Clinical study on the effect of ginkgo leaf extract and dipyridamole injection on early renal damage of essential hypertension. Medical Information. 23, 126-126

Li, L., 2006. Clinical Research Of Huangqi Injection In Treating Early kidney Lesion In Patients With Hypertension. Guangxi university of Chinese medicine.

Li, Q. H. and Guo, J. (2017). Effect of Tanshinone Injection Combined with Valsartan on Patients with Hypertensive Renal Damage. Xinjiang Medical Journal. 47, 1304-1305+1308

Li, T. (2016). Effect of tanshinone combined with valsartan on hypertensive nephropathy. The World Clinical Medicine. 10, 88

Li, Y. J. (2014). Clinical observation of tanshinone injection combined with valsartan in treating 115 cases of hypertensive renal damage. Chinese Journal of Ethnomedicine and Ethnopharmacy. 23, 57-58

Liu, F. (2012). Clinical observation of breviscapine combined with benazepril in the treatment of hypertensive renal damage. Chinese Journal of Practical Medicine. 39, 84-85

Liu, W. (2018). Therapeutic effect of Salvia miltiorrhiza polyphenolates combined with Valsartan on hypertensive Nephropathy. Prevention and Treatment of Cardiovascular Disease. 8, 8-9

Liu, W. H. (2012). Observation on the curative effect of combined traditional Chinese and western medicine on senile hypertensive renal damage. Chinese and Foreign Medical Research. 10, 50-51

Liu, Y. D. (2015). Clinical study of tanshinone in the treatment of hypertensive nephropathy. Abstract Edition: Medicine and Health. 0, 24

Liu, Z. F. (2018). Clinical characteristics of hypertensive nephropathy and evaluation of tanshinone injection combined with valsartan in treatment. Cardiovascular Disease Electronic Journal of Integrated Traditional Chinese and Western Medicine. 6, 74

Luo, J. (2014). Observation on the curative effect of combined traditional Chinese and western medicine on renal hypertension. Guiding Journal of Traditional Chinese Medicine and Pharmacy. 20, 19-20

Ma, H. H. (2018). Clinical effect of breviscapine injection on patients with hypertensive nephropathy. Medical Equipment. 31, 133-134

Ma, W. T.,Nie, F.,Sun, G. Q. and Song, G. Y. (2017). Effect of tanshinone combined with valsartan therapy on the renal injury and endothelial injury in patients with hypertensive nephropathy. Journal of Hainan Medical University. 23, 1059-1062

Meng, X. P. (2014). Clinical research of traditional Chinese medicine injection in treatment of early hypertensive nephropathy and its effects on renal function. Medical Journal of Chinese People's Health. 26, 15-16

Peng, J.,Cui, J.,Cheng, Y. G.,Wei, Q.,Wang, L. and Chen, G. (2013). Observation of curative effect of Danhong injection on early hypertensive nephropathy. Journal of Hunan University of Chinese Medicine. 33, 12-13

Qiao, S. Q. (2015). Clinical observation on treating hypertensive nephropathy with Dengzhanhua Su injection. Clinical Journal of Chinese Medicine. 07, 13-15

Qiu, S. J. (2015). Effect of tanshinone injection combined with valsartan in the treatment of hypertensive nephropathy. Abstract Edition: Medicine and Health. 0, 203

Ren, W. and Wu, H. K. (2006). Clinical observation on the treatment of hypertensive nephropathy with integrated traditional Chinese and western medicine. Modern Traditional Chinese Medicine. 26, 35-36

Shi, H. B.,Li, Y. F.,Xu, J. H.,Li, S.,Liu, J. G. and Jin, W. (2020). Effects of tanshinone IIA sodium sulfonate injection combined with Irbesartan on urinary NAG, β2-MG, serum PICP and PCX levels in patients with hypertensive complicated by kidney injury. Health Medicine Research and Practice. 17, 45-49

Tang, G. C.,Mo, Y. S.,Chen, W. and Lu, G. C. (2006). Protective effect of astragalus on early renal damage in hypertension. Modern Journal of Integrated Traditional Chinese and Western Medicine. 15, 26-27

Tian, Z. S.,Sun, Y. and Kong, X. F. (2019). Clinical effect analysis of salvianolate combined with valsartan in the treatment of hypertensive nephropathy. Yiyao Qianyan. 09, 50-51

Tu, M. S. (2017). Clinical observation of valsartan combined with tanshinone injection in the treatment of 64 cases of hypertensive renal damage. Guide of China Medicine. 15, 3+5

Wang, L. (2018). Clinical Evaluation on Salvianolate Combined with Valsartan in the Treatment of Hypertensive Nephropathy in 45 Cases. China Pharmaceuticals. 27, 44-46

Wang, L. L., 2015. The protective effect on hypertensive nephropathy with combination of salvianolate and valsartan. Hebei Medical University.

Wang, L. L.,Pan, X. and Wei, Z. F. (2015). Clinical observation of Salvia miltiorrhiza polyphenolates combined with Valsartan in the treatment of hypertensive Nephropathy. Shanxi Medical Journal. 44, 2901-2903

Wang, L. P. (2014). Clinical observation of Danhong injection in the treatment of early renal damage in senile hypertension. Inner Mongolia Journal of Traditional Chinese Medicine. 33, 27

Wang, Q.,Li, C. and Zhou, X. H. (2016). Analysis of therapeutic effect of tanshinone injection combined with valsartan in hypertensive nephropathy. Journal of Today Health. 15, 136. doi:10.3969/j.issn.1671-5160.2016.07.126

Wang, W. L. and Lan, D. L., 2012. Observation of therapeutic effect of breviscapine combined with lisinopril on hypertensive nephropathy. 2012 Zhejiang Medical Association Clinical Pharmacy Academic Annual Meeting and the 10th Anniversary Celebration Conference of Hospital Pharmaceutical Administration Quality Control Center and Clinical Pharmacy Branch, Hangzhou, Zhejiang, China, p. 3.

Wang, X. F.,Bu, S. Y. and Wu, Y. L. (2014). Effect of ginkgo leaf extract and dipyridamole injection on the renal function and the blood lipid of patients with hypertension complicated with renal insufficiency. China Modern Doctor. 52, 33-35

Wang, X. L. (2016). Curative efficacy of adjuvant therapy of Danhong injection in treating hypertensive nephropathy and its effect on urine protein and hemorheology. China Journal of Modern Medicine. 26, 124-127

Wang, X. Y. (2010). 80 cases of senile hypertensive renal damage treat with integrated traditional Chinese and western medicine. World Journal of Integrated Traditional and Western Medicine. 05, 966-968

Wei, L. and Tan, J. (2005). Clinical observation on Breviscapine in treating hypertension patients complicated with micro-albuminuria of renal impairment. Chinese Journal of Integrative Medicine. 11

Wu, X. L. (2013). Clinical observation of tanshinone in treating hypertensive nephropathy. China rural health. 0, 209

Wu, X. S. and Zhao, M. (2007). Observation of curative effect of astragalus injection on renal damage of essential hypertension. The Medical Forum. 11, 717-718

Xin, S. P. and Ren, A. H. (2013). Observation of Danhong Injection in Treating 30 Cases of Senile Hypertension with Early Renal Damage. Zhejiang Journal of Traditional Chinese Medicine. 48, 698

Xin, X. H. (2015). Pharmacological analysis of Danhong injection in the treatment of early renal damage in senile hypertension. World Latest Medicine Information. 15, 172-173

Xu, G. H.,Yuan, L.,Li, Y.,Xie, P.,Zhao, J. Y. and Chen, Y. H. (2008). Clinical observation of Astragalus Injection in treatment of renal injury in patients with primary hypertension. Journal of Integrative Medicine. 06, 530-532

Xu, Y. Q. (2015). Efficacy analysis of astragalus injection combined with irbesartan in the treatment of hypertensive nephropathy. World Latest Medicine Information. 15, 51-52

Yang, J. Y. (2015). Clinical observation ontreatment of primary hypertension with albuminuria by astragalus injection combined with hydrazia. Clinical Journal of Chinese Medicine. 07, 29-30

Yao, G. L.,Gui, B. L.,Ma, L. Q. and Zhang, X. Y. (2002). Effect of Astragalus Injection on Reducing Urinary Protein Content in Hypertensive Renal Damage. Shaanxi Medical Journal. 31, 341-343

Ye, B. (2009). Clinical analysis of breviscapine in treatment of hypertensive nephropathy. China Modern Medicine. 16, 64-65

Ye, H. and Zhang, Y. (2011). Clinical study of ginkgo leaf extract and dipyridamole injection combined with benazepril in the treatment of renal damage in elderly patients with essential hypertension. Strait Pharmaceutical Journal. 23, 137-138

Yu, H. T. (2014). Clinical study of tanshinone in treating hypertensive nephropathy. FamiIy psychological doctor. 0, 239+237

Zhang, Y.,Wang, J. S. and Zhang, L. M. (2004). Effect of breviscapine combined with lisinopril on urinary microalbumin in patients with hypertension. Hunan Journal of Traditional Chinese Medicine. 20, 12-13

Zhao, F. (2017). Study on curative effect of astragalus injection combined with irbesartan on hypertensive nephropathy. Shaanxi Journal of Traditional Chinese Medicine. 38, 51-52

Zhao, Q. and Dong, G. (2016). Effect of breviscapine on serum fibrosis and arterial elasticity indexes in patients with hypertensive nephropathy. Chinese Journal of Biochemical and Pharmaceuticals. 36, 151-153

Zhao, Y. F.,Cui, S. F.,Liang, F. F.,Li, Y. R.,Ji, M. J. and Xie, G. F. (2015). Effects of Astragalus membranaceus combined with irbesartan on renal function and urinary protein in patients with hypertensive renal damage. Hainan Medical Journal. 26, 1028-1030

Zhao, Y. J. and Jing, Z. X. (2015). Effect of Huangqi Injection on renal injury due to primary hypertension. Chinese Journal of Evidence-Based Cardiovascular Medicine. 07, 248-250

Zheng, X. L. (2006). Observation of therapeutic effect of breviscapine combined with western medicine on hypertensive nephropathy. Journal of Practical Traditional Chinese Medicine. 22, 144-145

Zhu, H. W.,Zhu, M. and Gao, J. D. (2011). Clinical study of hypertensive nephropathy treated with tanshinone. Journal of Internal Medicine Concepts & Practice. 06, 204-207

Zhu, Q. Q. (2015). Feasibility and value of tanshinone injection combined with valsartan in the treatment of hypertensive nephropathy. Abstract Edition: Medicine and Health. 0, 192

Zou, H. L.,Zhan, J.,Wang, Y. J. and Chen, R. G. (2013). Clinical Observation on Tanshinone and valsartan for treatment hypertensive nephropathy. Chinese Journal of Integrative Medicine on Cardio-Cerebrovascular Disease. 11, 1303-1304

# S File 4 PRISMA Checklist

| **Section and Topic** | **Item #** | **Checklist item** | **Location where item is reported** |
| --- | --- | --- | --- |
| **TITLE** | | |  |
| Title | 1 | Identify the report as a systematic review. | 1 |
| **ABSTRACT** | | |  |
| Abstract | 2 | See the PRISMA 2020 for Abstracts checklist. | 3 |
| **INTRODUCTION** | | |  |
| Rationale | 3 | Describe the rationale for the review in the context of existing knowledge. | 5 |
| Objectives | 4 | Provide an explicit statement of the objective(s) or question(s) the review addresses. | 6 |
| **METHODS** | | |  |
| Eligibility criteria | 5 | Specify the inclusion and exclusion criteria for the review and how studies were grouped for the syntheses. | 7 |
| Information sources | 6 | Specify all databases, registers, websites, organisations, reference lists and other sources searched or consulted to identify studies. Specify the date when each source was last searched or consulted. | 8 |
| Search strategy | 7 | Present the full search strategies for all databases, registers and websites, including any filters and limits used. | 8 |
| Selection process | 8 | Specify the methods used to decide whether a study met the inclusion criteria of the review, including how many reviewers screened each record and each report retrieved, whether they worked independently, and if applicable, details of automation tools used in the process. | 9 |
| Data collection process | 9 | Specify the methods used to collect data from reports, including how many reviewers collected data from each report, whether they worked independently, any processes for obtaining or confirming data from study investigators, and if applicable, details of automation tools used in the process. | 9 |
| Data items | 10a | List and define all outcomes for which data were sought. Specify whether all results that were compatible with each outcome domain in each study were sought (e.g. for all measures, time points, analyses), and if not, the methods used to decide which results to collect. | 9 |
|  | 10b | List and define all other variables for which data were sought (e.g. participant and intervention characteristics, funding sources). Describe any assumptions made about any missing or unclear information. | 9 |
| Study risk of bias assessment | 11 | Specify the methods used to assess risk of bias in the included studies, including details of the tool(s) used, how many reviewers assessed each study and whether they worked independently, and if applicable, details of automation tools used in the process. | 9 |
| Effect measures | 12 | Specify for each outcome the effect measure(s) (e.g. risk ratio, mean difference) used in the synthesis or presentation of results. | 9 |
| Synthesis methods | 13a | Describe the processes used to decide which studies were eligible for each synthesis (e.g. tabulating the study intervention characteristics and comparing against the planned groups for each synthesis (item #5)). | 9 |
|  | 13b | Describe any methods required to prepare the data for presentation or synthesis, such as handling of missing summary statistics, or data conversions. | 10 |
|  | 13c | Describe any methods used to tabulate or visually display results of individual studies and syntheses. | 10 |
|  | 13d | Describe any methods used to synthesize results and provide a rationale for the choice(s). If meta-analysis was performed, describe the model(s), method(s) to identify the presence and extent of statistical heterogeneity, and software package(s) used. | 10 |
|  | 13e | Describe any methods used to explore possible causes of heterogeneity among study results (e.g. subgroup analysis, meta-regression). | 10 |
|  | 13f | Describe any sensitivity analyses conducted to assess robustness of the synthesized results. | 10 |
| Reporting bias assessment | 14 | Describe any methods used to assess risk of bias due to missing results in a synthesis (arising from reporting biases). | 10 |
| Certainty assessment | 15 | Describe any methods used to assess certainty (or confidence) in the body of evidence for an outcome. |  |
| **RESULTS** | | |  |
| Study selection | 16a | Describe the results of the search and selection process, from the number of records identified in the search to the number of studies included in the review, ideally using a flow diagram. | 10 |
|  | 16b | Cite studies that might appear to meet the inclusion criteria, but which were excluded, and explain why they were excluded. | 11 |
| Study characteristics | 17 | Cite each included study and present its characteristics. | 11 |
| Risk of bias in studies | 18 | Present assessments of risk of bias for each included study. | 11 |
| Results of individual studies | 19 | For all outcomes, present, for each study: (a) summary statistics for each group (where appropriate) and (b) an effect estimate and its precision (e.g. confidence/credible interval), ideally using structured tables or plots. | 11 |
| Results of syntheses | 20a | For each synthesis, briefly summarise the characteristics and risk of bias among contributing studies. | 12 |
|  | 20b | Present results of all statistical syntheses conducted. If meta-analysis was done, present for each the summary estimate and its precision (e.g. confidence/credible interval) and measures of statistical heterogeneity. If comparing groups, describe the direction of the effect. | 13 |
|  | 20c | Present results of all investigations of possible causes of heterogeneity among study results. | 13 |
|  | 20d | Present results of all sensitivity analyses conducted to assess the robustness of the synthesized results. | 13 |
| Reporting biases | 21 | Present assessments of risk of bias due to missing results (arising from reporting biases) for each synthesis assessed. | 11 |
| Certainty of evidence | 22 | Present assessments of certainty (or confidence) in the body of evidence for each outcome assessed. |  |
| **DISCUSSION** | | |  |
| Discussion | 23a | Provide a general interpretation of the results in the context of other evidence. | 15 |
|  | 23b | Discuss any limitations of the evidence included in the review. | 17 |
|  | 23c | Discuss any limitations of the review processes used. | 17 |
|  | 23d | Discuss implications of the results for practice, policy, and future research. | 17 |
| **OTHER INFORMATION** | | |  |
| Registration and protocol | 24a | Provide registration information for the review, including register name and registration number, or state that the review was not registered. | 7 |
|  | 24b | Indicate where the review protocol can be accessed, or state that a protocol was not prepared. | 7 |
|  | 24c | Describe and explain any amendments to information provided at registration or in the protocol. | 7 |
| Support | 25 | Describe sources of financial or non-financial support for the review, and the role of the funders or sponsors in the review. | 19 |
| Competing interests | 26 | Declare any competing interests of review authors. | 19 |
| Availability of data, code and other materials | 27 | Report which of the following are publicly available and where they can be found: template data collection forms; data extracted from included studies; data used for all analyses; analytic code; any other materials used in the review. | 19 |
